# Regulation and Site-Specific Covalent Labeling of *NSUN2* via Genetic Encoding Expansion

**DOI:** 10.3390/genes12101488

**Published:** 2021-09-24

**Authors:** Jizhong Zhao, Hongmei Hu, Sheng Wang, Li Wang, Rui Wang

**Affiliations:** 1 The Hubei Key Laboratory of Natural Resource and Medicine, School of Pharmacy, Tongji Medical College, Huazhong University of Science and Technology, 13 Hangkong Road, Wuhan 430030, China; zhaojz1990@163.com (J.Z.); huhongmei0001@126.com (H.H.); D201881311@hust.edu.cn (S.W.); 2 Wuhan No.1 Hospital, Huazhong University of Science and Technology, 225 Zhongshan Avenue, Wuhan 430022, China; Wangli5033@sina.com

**Keywords:** RNA modification, m^5^C, NSUN enzyme, regulation, labeling

## Abstract

In living organisms, RNA regulates gene expression, cell migration, differentiation, and cell death. 5-Methylcytosine is a post-transcriptional RNA modification in a wide range of RNA species, including messenger RNAs. The addition of m^5^C to RNA cytosines is enabled by the *NSUN* enzyme family, a critical RNA methyltransferase. In this study, natural lysines modified with special groups were synthesized. Through two rounds of positive screening and one round of negative screening, we evaluated and identified the *MbPylRS*-tRNA_CUA_ unnatural lysine substitution system, which specifically recognizes lysine with a defined group. Moreover, non-natural lysine substitution at C271 of *NSUN2* active site and the subsequent fluorescent labeling was realized through the click reaction. Then, the function of the *NSUN2* mutant and its upregulated *CDK1* gene as well as its effect on cell proliferation were evaluated. Efficient labeling and regulation of *NSUN2* was achieved, laying the basis for further studies on the function and regulatory mechanism of upregulated genes.

## 1. Introduction

In living organisms, ribonucleic acids regulate gene expression, cell migration, differentiation, and cell death [1,2]. Besides the four canonical nucleotides, over 150 chemical modifications in endogenous nucleic acids facilitate their diversified structures and functions [3,4,5]. To achieve the regulation purpose, RNA modifying enzymes play essential roles, adding a wide range of chemical modifications into target RNAs. Methylation is heavily associated with intrinsic RNAs in various species [6,7,8,9,10,11,12]. Compared to m^6^A, 5-methylcytosine (m^5^C) is a less common modification in mammalian animals that has increasingly been evaluated in recent years. The m^5^C modification is performed by various enzymes, including *NOL1/NOP2/SUN* domain (*NSUN*) family [13,14,15]. m^5^C is a post-transcriptional RNA modification in various RNA species, including messenger RNAs and transfer RNA among others [11]. The addition of m^5^C to RNA is enabled using the *NSUN* family of enzymes and the DNA methyltransferase, *DNMT2*, in mammalian cells. *NSUN2* is a critical RNA methyltransferase for adding m^5^C to mRNA. Yang et al. revealed that m^5^C modification is enriched in the CG-rich motif [14,15], which is located down-stream of translation initiation sites and has conserved features across mammalian transcriptomes. Moreover, m^5^C is recognized by the mRNA export adaptor, *ALYREF*, as shown by in vitro and in vivo studies, where *NSUN2* modulated nuclear-cytoplasmic shuttling of *ALYREF*, RNA-binding affinity, and associated mRNA export [13]. Based on the roles of *NSUN2* enzymes in m^5^C-associated RNA biological activities in living organisms, including cellular proliferation, senescence, migration, differentiation, mRNA nuclear export, enhanced mRNA translation, tRNA stabilization, and cleavage [16], achieving site-specific labeling and modulation of *NSUN2* is, therefore, of high importance. However, precise regulation of nuclear-cytoplasmic shuttling of endogenous RNAs by manipulating the activity of *NSUN2* remains elusive.

**Figure 1:**
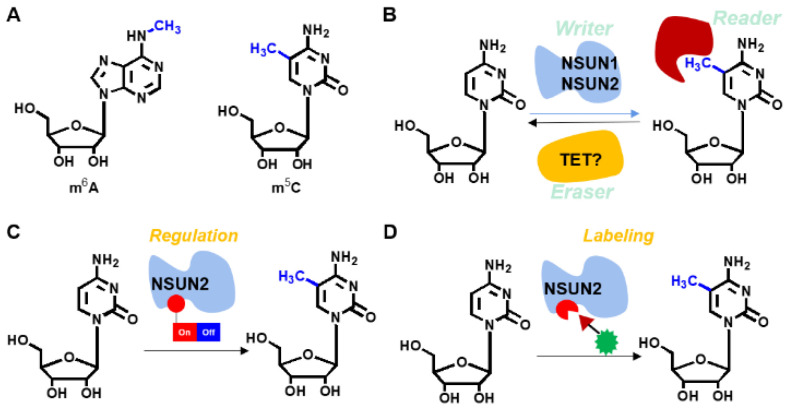
Investigations of RNA m^5^C modification. (**A**) Representative modifications in RNA, including m^6^A and m^5^C, among others. (**B**) m^5^C involved enzyme, including the ‘writer’ enzyme, *NSUN2*. (**C**) Strategy of a switch precisely setting on the *NSUN2* that regulates RNA methyl modification on cytosine 5 position by a gene encoding expansion technique. (**D**) Site-specific labeling of *NSUN2* was achieved by gene encoding expansions and bio-orthogonal click reaction.

The genetic encoding expansion technique developed by Schultz et al. has been successfully applied in mammals as a powerful tool in molecular biology. It has been used in identification of PPIs, regulation of proteins and RNAs as well as in drug discoveries among others [17,18,19,20,21,22]. In recent years, achievements have been made in protein regulation. For instance, Chen et al. used genetic encoding expansion techniques manipulate the functions of various enzymes, including *FTO*, *luciferase*, and *KRAS* [22]. Based on our previous study [23], we installed the non-canonical PBBK into the critical site of Cas9 endonuclease through genetic encoding expansion, achieving a precise regulation of CRISPR-Cas9 gene editing. Therefore, we postulate that *NSUN2* can be labeled and regulated through the genetic encoding expansion technique (Figure 1). Moreover, if the defined functionality is simultaneously installed, regulation of m^5^C levels on specific RNAs in a spatiotemporal manner and further site-specific labeling of *NUSN2* can be achieved. Therefore, this study aimed at evaluating the intrinsic nature of the m^5^C modifying enzyme, *NSUN2*. Moreover, site-specific labeling and modulation of *NSUN2* besides the upregulated genes was also clarified.

## 2. Materials and Methods

All chemicals were purchased from commercially available sources otherwise stated including Innochem (Beijing, China), Aladdin (Shanghai, China), Ark (Shanghai, China), TCI (Shanghai, China), Sigma-Aldrich Inc. (Shanghai, China). All solvents were used directly purchased from Innochem (Shanghai, China) without further purification. Buffers including phosphate-buffered saline (PBS) and Tris(hydroxymethyl) aminomethane (Tris) were purchased from Innochem (Beijing, China). 4-(2-Hydroxyethyl)piperazine-1-ethanesulfonic acid (HEPES, Cas # 7365-45-9), acrylamide and thiazolyl blue tetrazolium bromide (MTT, Cas # 298-93-1) were purchased from Sigma-Aldrich (Shanghai, China). 

NMR were performed on a *Bruker AM-400 spectrometer*. Mass spectra was performed on *Advion Expression L* (Bohui Innovation Biotechnology, Beijing, China) using electrospray ionization (ESI). UV spectra was performed on *Perkin Elmer Lambda 365* (Minden, German). Gel Imaging was performed using *Pharos FX Molecular Imager* (Bio-Rad, Hercules, CA, USA). Confocal microscope images were analyzed on *Zeiss LSM780*. Flow cytometry were analyzed on an *LSR-II Flow Cytometer* (BD Biosciences, Franklin Lakes, NJ, USA) and data were analyzed using *FlowJo* software (Tree Star, HongKong, China).

Synthesis of substrate S1. 2-Nitrobenzyl alcohol (500.0 mg, 3.3 mmol) was dissolved in THF (7.0 mL), containing Na_2_CO_3_ (345.5 mg, 3.3 mmol), and cooled to 0 °C. Triphosgene (967.4 mg, 3.3 mmol) was added to this solution and the reaction was continued to be stirred at r.t. for 12 h. The reaction was filtered, and the volatiles were subsequently evaporated without heating and the residue dried under vacuum to give carbamate intermediate in quantitative yield (702.8 mg, 3.3 mmol). Carbamate intermediate (702.8 mg, 3.3 mmol) was added to a solution of Boc-l-lysine (840.6 mg, 3.6 mmol) in a mixture of THF and 1.0 M NaOH (1: 4, 14.0 mL) at 0 °C. The reaction was stirred at r.t. for 12 h. The aqueous layer was washed with Et_2_O (5.0 mL) and subsequently acidified with ice-cold 1.0 M HCl (20.0 mL) to pH = 1 and was extracted with ethyl acetate (30 mL). The organic layer was dried over Na_2_SO_4_, filtered, and the volatiles were evaporated to give Boc-S1 (1.2 g) in 87% yield. ^1^H-NMR (400 MHz, CDCl_3_) δ 8.06 (d, J = 8.0 Hz, 1H), 7.73–7.53 (m, 2H), 7.53–7.35 (m, 1H), 5.43 (s, 2H), 4.31–4.27 (m, 1H), 3.21–3.17 (m, 2H), 1.85–1.80 (m, 2H), 1.71–1.67 (m, 2H), 1.57–1.52 (m, 2H), 1.42 (s, 9H). ^13^C-NMR (101 MHz, CDCl_3_) δ 176.15, 156.29, 147.04, 137.52, 133.84, 133.24, 128.42, 127.85, 124.77, 79.93, 63.07, 61.48, 40.65, 31.91, 28.23, 22.41. MS (ESI): [M + Na] ^+^ = 447.8; HRMS (EI): Calculated for C_19_H_27_N_3_O_8_ [M + Na] ^+^: 448.1696; Found: 448.1754.

Overall protocol for unnatural lysine substitution.

Step 1.1. Screen specific *MbPylRS*.
Step 1.2. Construction of a dual fluorescence reporter system for unnatural amino acid substitution detection. Details are presented in Supplementary Material.
Step 1.3. Replacement of unnatural amino acids in the active site of *NSUN2*.

Through the above experiments, the construction and screening of the unnatural amino acid substitution system for the above four lysine derivatives in eukaryotic cells have been completed, and then the unnatural amino acid substitution of the active site C271 in *NSUN2* will be further implemented. C271 is an important active site of *NSUN2*, responsible for the separation of *NSUN2* from the substrate after catalysis.

Step 1.4. Western blot detection of the effect of unnatural amino acid substitution at C271 of *NSUN2* on protein expression.

First, the eukaryotic expression system of *NSUN2* was constructed as shown. The C271 site of the *NSUN2* gene sequence was mutated to a TAG amber codon. The C-terminal of *NSUN2* has an EGFP fluorescent protein tag. *NSUN2-EGFP* eukaryotic expression system was transfected into *MbPylRS*-tRNA_CUA_ expressing cells. The cells were cultured in a DMEM medium containing different lysine derivatives. After 24 h, the cells were lysed, and the total cell protein was extracted. The protein expression of *NSUN2* was detected by Western blot. The experimental results show that the expression of *NSUN2* C271 mutant protein can be detected in the presence of S1–S4. 

In HeLa cells expressing the *MbPylRS*-tRNA_CUA_ system, they were cultured in a DMEM medium containing S1–S4 (2.0 mM) synthetic lysine. After 24 h, the cells were lysed, and the total protein was extracted. The protein expression was detected by Western blot. Wild-type HeLa cells were used as a negative control, and HeLa cells transfected with *NSUN2* overexpression plasmid were used as a positive control.

Bio-orthogonal reaction to label *NSUN2* and detect its subcellular localization.

Through Western blot detection, it is found that the unnatural amino acid substitution of C271 at the mutation site of *NSUN2* can be efficiently realized in the presence of S3. Next, the click reaction was used to label S3 at the C271 site of *NSUN2* with Cy5 fluorescent dye, and the subcellular localization of *NSUN2* could be tracked. To further test whether this protein labeling method is accurate, the *EGFP* fusion protein of *NSUN2* was constructed as described in the previous section. Detecting the co-localization of Cy5 (red fluorescence) and *EGFP* (green fluorescence) can detect the subcellular localization of *NSUN2*. In addition, in order to improve the expression efficiency of *NSUN2*, the concentration of S3 was further optimized. Cells were cultured in a DMEM medium containing 3.0 mM, 2.0 mM, and 1.0 mM S3, respectively, and then stained with Cy5 to detect protein localization under a fluorescence microscope. The experimental results show that with the continuous increase of Lys-N_3_ (S3) addition level, the replacement efficiency of unnatural amino acids in the process of *NSUN*2 protein expression also gradually increases, and the positioning of green fluorescent protein and red fluorescence shows that there are two kinds of fluorescence. The phenomenon of co-localization also shows that Lys-N_3_ (S3) has achieved amino acid substitution at position C271 of *NSUN2*. 

In the HeLa cells expressing the *MbPylRS*-tRNA_CUA_ unnatural amino acid substitution system and the *NSUN2-EGFP* C271TAG mutation system, the cells were cultured in a DMEM medium containing different concentrations of S3 (1.0, 2.0, 3.0 mM) for 24 h, and then reacted with Cy5 and Lys-N_3_ (S3) via the click reaction to label *NSUN2* with unnatural amino acids. *NSUN2* itself has a *EGFP* label, and the subcellular localization of the two fluorescence could be detected by fluorescence microscope to reflect the unnatural amino acid replacement efficiency and subcellular localization of *NSUN2*.

## 3. Results

**Figure 2:**
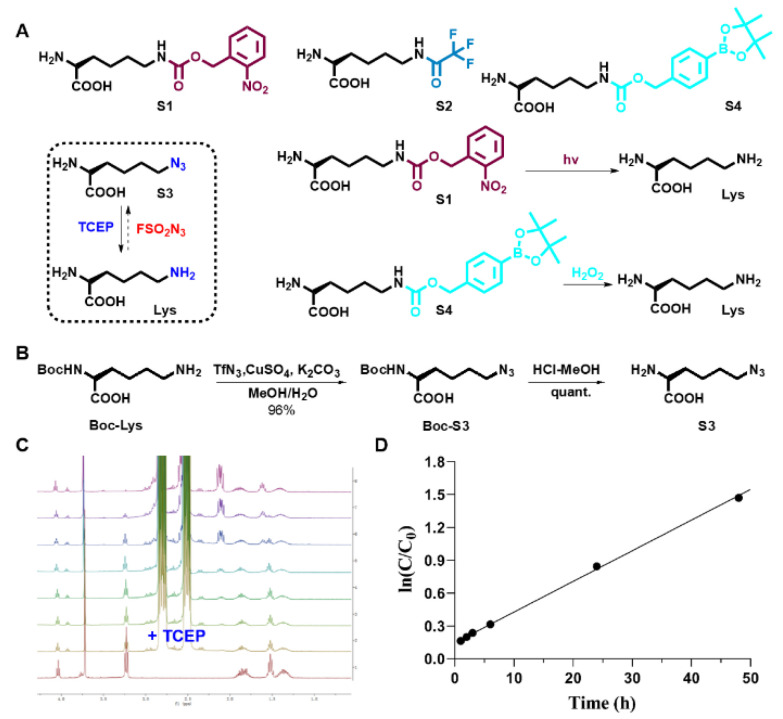
Investigations of lysine analogue utilizations in *NSUN2* regulation studies. (**A**) Substrates (S1–S4) and their small molecule or UV-light induced reversal methods. (**B**) Synthetic details of 6-azido lysine (S3). (**C**) Illustration of Staudinger reduction of S3 with TCEP (10 eq., in D_2_O) by use of in situ proton NMR experiment. (**D**) Analysis of data for the reaction of S3 with TCEP (10 eq.). Plotting time vs ln(C/C_0_) yields a straight line; therefore, it was identified to be a pseudo-first order dynamic where S3 (20 mM), TCEP (200 mM) in D_2_O (0.6 mL), with a slope 0.02798, and a half time t_1/2_= 24.8 min. C represents S3 concentration at a defined time, while C_0_ represents initial concentration of S3. TCEP = Tris (2-carboxyethyl)-phosphine. S1–S3 are previously reported compounds, S4 is a compound recently synthesized by us.

To establish the feasibility of our method, we synthesized four lysine substrates (Supplementary Figures S1–S14, Schemes S1–S4). As shown in Figure 2, the four lysine substrates were synthesized and evaluated by NMR and mass spectroscopy (Figure 2A). S3 was obtained from commercially available Boc-protected *L*-lysine through two linear steps. A yield of over 95% was obtained (Figure 2B). The four lysine analogs can be transformed into their native forms using established methods [23,24,25,26]. For instance, substrate S1 was restored to lysine by UV (365 nm)-light within 5 min [24]. Substrate S2 was transformed to its untreated form through basic hydrolysis [25] while substrate S4 was rescued into lysine by the signaling molecule, hydrogen peroxide, as previously reported. [23] Additionally, substrate S3 was restored to its native form through rapid addition of tricarboxyethyl phosphine (TCEP) [26]. Kinetic investigation of the S3 reaction using TCEP revealed a pseudo-first order with a half time of 24.8 min (Figure 2C,D and Supplementary Figure S15, Table S1, Chart S1).

Construction of a site-specific unnatural amino acid substitution system. We further determined whether the substrates (S1–S4) can be site-specifically incorporated into the *NSUN2* enzyme (Supplementary Figure S17). Based on the structure of the m^5^C modification enzyme, *NSUN6* [27], cysteine at position 271 plays an important role in RNA m^5^C modifications. However, the crystal structure of *NSUN2* has not been established (Supplementary Figure S16). By screening the *NSUN2* mutant that had been randomly incorporated with our lysine analogs (i.e., our substrates S1–S4), regulation of *NSUN2* enzyme activity was achieved, since they were caged. As for specific substrate S3, terminal 6-amino group was transformed to azido group, compared to lysine. As postulated, the *NSUN2* activity could be restored if the Staudinger reaction was applied to reduce the azido group to the amine functionality [27]. Moreover, bio-orthogonal reaction of the azido group with various alkynes (attached with fluorescence or biotinylated functionality) was performed, which realized fluorescent labeling or chemical pulldown.

Then, we screened tRNA transferases (*MbPylRS*) with abilities to specifically recognize lysine derivatives with defined modified groups.

Screening of specific *MbPylRS*. First, the mutation library was obtained through random mutations of the *MbPylRS* active site. Random mutations were performed in six *MbPylRS* sites, including L266, L270, Y271, L274, C313, and W383, with 10^8^ mutants being theoretically produced. Then, *MbPylRS* mutants with the ability to specifically recognize lysine derivatives (S1–S4) were screened from the mutant library. To facilitate the screening process, we constructed two screening systems [18,19,20]. Through one positive and two negative screening processes, the *MbPylRS*-tRNA_CUA_ system with specific recognition and coding abilities for target lysine derivatives (S1–S4) was identified. Therefore, we achieved efficient replacements of unnatural lysines (S1–S4) at *NSUN2* important sites (Supplementary Figure S19–S21).

**Figure 3:**
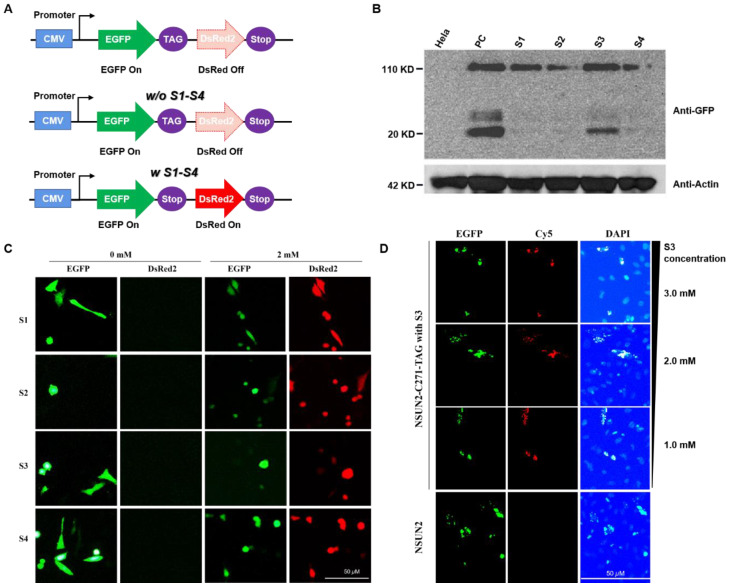
Fluorescent labeling of *NSUN2*. (**A**) Schematic presentation of the construction of the dual fluorescent reporter system. (**B**) Detection of replacement efficiencies using various unnatural amino acids by monitoring expressions of the *NSUN2* protein through Western blot assays. HeLa cells expressing the *MbPylRS*-tRNA_CUA_ system were cultured in DMEM medium containing S1–S4 (2.0 mM). Briefly, HeLa cells were lysed and total proteins extracted after 24 h. Protein expression levels were detected by Western blot. HeLa cells transfected with the *NSUN2* overexpression plasmid were set as the positive control group. S1–S4 exhibited great replacement efficiencies (110 KD band). (**C**) Detection ofthe recognizing of the screened MbPylRS-tRNA_CUA_ to the specific amino acid derivatives. In the absence of S1–S4 (0 mM, left), only green fluorescent protein signals were detected. In the presence of S1–S4 (2.0 mM), both EGFP and DsRed protein signals were recorded (right). (**D**) HeLa cells expressing the *MbPylRS*-tRNA_CUA_ amino acid substitution and *GFP-NSUN2* C271TAG mutation systems were cultured in DMEM medium supplemented with different concentrations of S3 (1.0, 2.0, and 3.0 mM) and incubated for 24 h. Then, DBCO-Cy5 (50 µM) was added to react with lys-N_3_ (S3) in *NSUN2*. Subcellular localization of *NSUN2* was identified by dual-color fluorescence imaging when S3 (as low as 1.0 mM) was applied. Scale bar: 50 µM.

Construction of dual fluorescence reporter system for detection of unnatural lysine substitution. To determine whether screening of unnatural lysine substitution system is feasible, a dual fluorescence reporter system was established. HeLa cells expressing *MbPylRS*-tRNA_CUA_ and dual fluorescent reporter genes were cultured in a DMEM medium with specific lysine derivatives (S1–S4). After 24 h, expressions of fluorescent proteins were detected. *MbPylRS*-tRNA_CUA_, which specifically recognized the four lysine derivatives (S1–S4), was successfully selected after three rounds of screening. As shown in Figure 3C, expression of the red fluorescent proteins failed in the absence of S1–S4, while expressions of the two fluorescent proteins were detected in the presence of S1–S4. The results indicated that *MbPylRS*-tRNA_CUA_ could recognize specific unnatural lysine and code the amber codon, implying efficient replacement of lysine with defined S1–S4 on this system.

Substitution with unnatural amino acids at the important site of *NSUN2*. The C271 is a critical site of *NSUN4* (Supplementary Figure S16). It is important for separation of *NSUN* from the substrate after catalytic activity completion [13,14,15]. To establish the feasibility of substitution with unnatural lysine on the C271 site, we constructed a C271 *NSUN2* mutant in eukaryotic cells. First, the eukaryotic expression system of *NSUN2* was constructed; then, the C271 of *NSUN2* gene sequence was mutated into the TAG amber codon, and the C-terminal of *NSUN2* labeled with the *EGFP* fluorescent protein. In *MbPylRS*-tRNA_CUA_ expression cells, the *NSUN2-EGFP* eukaryotic expression system was transiently stained. Cells were cultured in DMEM medium supplemented with various lysine derivatives (S1–S4). After 24 h, cells were lysed and total proteins extracted. Protein expressions of *NSUN2* were further analyzed by Western blot assays. We confirmed that expression of the *NSUN2* C271 mutant protein could be detected in the presence of S1–S4 (Figure 3B). Expression levels of the *NSUN2* protein in the presence of S1 or S3 were relatively high (Figure 3B), whereas expressions of *NSUN2* protein in the presence of S2 and S4 were much lower level compared to S1 or S3 (Supplementary Figures S22 and S23). In addition to the desired target expression, a 20 KD band was expressed, it was attributed to the co-transfection process.

Detection of *NSUN2* using S3 (lys-N_3_) by a Staudinger reaction with Cy5 in eukaryotic cells. To determine whether *NSUN2* can be located and detected in subcellular regions, click reaction was performed based on azido functionality on *NSUN2* (Supplementary Schemes S5 and S6 and Figure S18). The unnatural lysine substitution of *NSUN2* mutation C271 was efficiently realized in the presence of S3. Moreover, *NSUN2* with S3 incorporated on the C271 site was labeled with the DBCO-Cy5 fluorescent dye through the click reaction. As such, subcellular location of the *NSUN2* mutant was established (Figure 3D, red dot). To assess the accuracy of this protein labeling method, the *EGFP* fusion protein of *NSUN2* was constructed. Subcellular localization of *NSUN2* was detected by monitoring co-localizations of DBCO-Cy5 (red fluorescence) and *EGFP* (green fluorescence) (Figure 3D). To enhance expression efficacies of *NSUN2*, S3 concentrations were optimized. Cells were cultured in DMEM medium with 1.0 mM, 2.0 mM, and 3.0 mM of S3. Respectively. Then, protein localization was detected by fluorescence microscopy after staining with DBCO-Cy5 (Supplementary Schemes S5 and S6). There was an enhanced substitution efficacy of unnatural lysine during NSUN2 protein expression with elevated concentrations of S3 (lys-N_3_). Through the detection of localizations of the green fluorescent protein and the red fluorescence, dual fluorescence were co-located, indicating efficient incorporation of S3 at the NSUN2 C271 site, displaying a potential for real-time tracking of NSUN2. Our findings show that biological orthogonal reactions of unnatural lysine with the azido group together with genetic encoding expansions enable specific NSUN2 protein labeling and tracking (Figure 3C,D).

**Figure 4:**
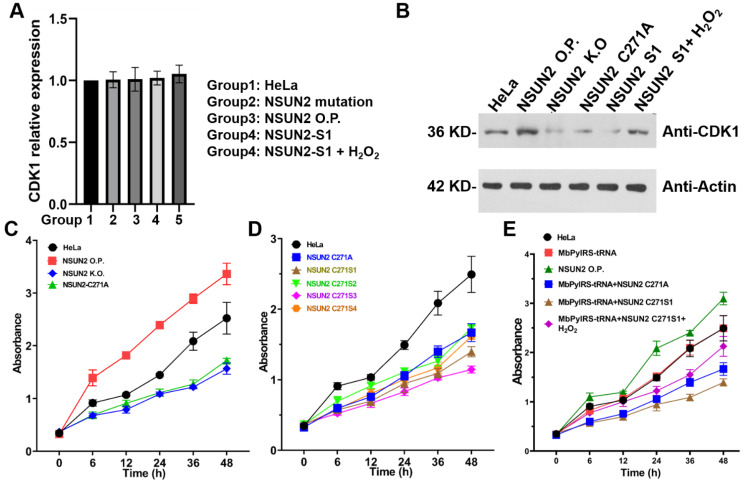
Investigations of *NSUN2* mutants towards cells activities. (**A**) Detection of the effect of *NSUN2* overexpression, knockout, and C271 mutated to S1 and C271S1 restored to natural lysine on downstream gene *CDK1* in HeLa cells via RT-PCR agarose gel electrophoresis. The experimental results show that *NSUN2* has no significant effect on the transcription level of *CDK1*. (**B**) Detection of the effect of *NSUN2* overexpression, knockout, C271 mutated to S1 and C271S1 restored to natural lysine on downstream gene *CDK1* in HeLa cells via Western blotting assays. (**C**) The effect of *NSUN2* C271A mutation and C271 mutated with S1–S4 on cell proliferation activities, CCK8 was used to detect cell proliferation. The mutation of C271 site significantly inhibited the activity of *NSUN2*, and *NSUN2* C271 mutated with S3 (pink color) displayed the highest inhibition activity. (**D**) The effect of *NSUN2* overexpression, knockout, and active site mutation (C271A) in HeLa cells on cell proliferation. It was noted that the mutation of C271 site significantly inhibited the activity of *NSUN2*. (**E**) C271 mutation or replaced by S1 in *NSUN2* significantly inhibited HeLa cell proliferation ability. However, the proliferation ability was partly rescued when S1 was transformed into nature lysine by use of H_2_O_2_. *MyPylRS*- tRNA and *NSUN2* overexpression system were transfected into HeLa cells as control.

Effects of *NSUN2* active site mutations on function. To evaluate the effect of *NSUN2* C271 mutation on the activity of *NSUN2*, we investigated the mechanism of its upregulated gene. First, the effect of *NSUN2* C271 mutation to alanine was evaluated. As an RNA methyltransferase, the mutant on *NSUN2* dysregulates RNA methylation, affecting the function and stability of target RNA [28,29,30,31,32]. Therefore, we knocked out *NSUN2* in HeLa cells, then replenished the wild-type or C271 mutant of *NSUN2*. Proliferative abilities of HeLa cells were then detected using the CCK8 kit, while the effects of *NSUN2* on *CDK1* transcription levels were evaluated by RT-PCR. The *NSUN2* mutant had no significant effect on *CDK1* transcription levels (Figure 4A). Upon *NSUN2* knock-out, cell proliferation levels were significantly suppressed. When HeLa cells were supplemented with wild-type *NSUN2*, cell proliferation levels returned to the wild-type level [32]. Notably, when *NSUN2* C271 was mutated to alanine, cell proliferation levels were significantly sup-pressed, implying that the C271 mutation significantly inhibited NSUN2 activities (Figure 4B). 

Effects of unnatural lysine substitution on the function of *NSUN2*. Regulatory effects of *NSUN2* after replacement of C271 with unnatural lysine (S1–S4) were investigated. *MbPylRS*-tRNA_CUA_ unnatural lysine substitution system and *NSUN2*-C271TAG mutant were co-expressed in HeLa cells. Cells were cultured in a DMEM medium containing S1–S4, after which proliferative abilities of tumor cells were detected. Proliferation levels of cells with S1 or S3 substrates were significantly suppressed when compared to wild-type cells, but were significantly higher than for cells with substrates S2 or S4. Therefore, the system with S1 and S3 exhibited higher replacement efficiencies than the one with S2 and S4 at *NSUN2* C271 sites (Figure 4C).

## 4. Discussion and Conclusions

First, random mutation of the active site is used to obtain the *MbPylRS* random mutation library. Here, we mainly performed random mutations at six positions of *MbPylRS* L266, L270, Y271, L274, C313, W383, and theoretically produced 10^8^ mutants. From this mutant library, *MbPylRS* mutants that can specifically recognize lysine derivatives were selected. In order to facilitate screening, two screening systems were constructed next. The first screening system is a forward screening system, in which two sites in the tetracycline gene sequence are replaced with TAG amber codons, and in the presence of the *MbPylRS*-tRNA_CUA_ system, if the *MbPylRS* mutant can specifically recognize the band Lysine derivatives with specific modification groups can convert the amber stop codon into an intentional codon that can encode lysine derivatives. Thus, tetracycline can be expressed smoothly. In the medium containing tetracycline, the strain that can encode TAG can survive, otherwise it cannot survive. The second screening system is a negative screening system. In this system, Barnase ribonuclease is selected. As Barnase has strong cytotoxicity, cells cannot survive in strains expressing Barnase. Here, the two sites in Barnase were also mutated to amber codons. On the basis of positive screening, negative screening was performed here. The culture medium contained no specific lysine derivatives and contained *MbPylRS*-tRNA_CUA_ system and Barnase negative screening system strains, if the strains can survive, it indicates that *MbPylRS*-tRNA_CUA_ has specific recognition for specific lysine derivatives. However, if the strain is unable to survive, it indicates that Barnase is expressed, which means that *MbPylRS*-tRNA_CUA_ has no specific or poor specificity for the recognition of specific lysines, and can also recognize other amino acids. Through the above two screening systems, after two rounds of positive screening and one round of invisible screening, the *MbPylRS*-tRNA_CUA_ system with specific recognition and coding capabilities for target amino acid derivatives can be screened. Through this system, the *NSUN2* active site will be further realized. 

In order to test whether the screening of the unnatural amino acid replacement system is successful, a dual fluorescent reporter system was further constructed, and two amber codons were inserted between *EGFP* and *DsRed*, and the transcription and expression of the two fluorescent genes shared the same set of initiating factors. The HeLa cells expressing *MbPylRS*-tRNA_CUA_ and dual fluorescent reporter gene system were cultured in a DMEM medium containing specific lysine derivatives, and the expression status of fluorescent protein was detected 24 h later. Due to the existence of amber codons, if the unnatural amino acid substitution system can successfully recognize specific lysine derivatives and encode amber codons, the expression of two fluorescent proteins can be detected simultaneously under a fluorescence microscope. To realize the recognition of lysine derivatives, or the encoding of amber codons cannot be realized, the expression of *DsRed* cannot be further realized after the expression of *EGFP*. Therefore, only green fluorescence can be detected in this case. The experimental results show that after three rounds of screening, *MbPylRS*-tRNA_CUA_, which can specifically recognize the above four lysine derivatives, was successfully screened, and the unnatural amino acid substitution of the target protein specific target in eukaryotic cells was successfully achieved. Simultaneous transfection of the dual fluorescence reporter system and unnatural amino acid substitution system in HeLa cells was performed, followed by incubation for 24 h in a DMEM medium without and with S1–S4 (2.0 mM). Then, the expression of fluorescent protein was detected under an inverted fluorescence microscope.

As an m^5^C methyltransferase, studies on the function of *NSUN2* are in their infancy stage—specifically, regulation of its RNA substrate [11]. Regulation of important sites of *NSUN2* may be an efficient approach for modulating the function of these enzymes. There are two active sites of *NSUN2*, including C321 and C271. C321 is responsible for methyl transfer while C271 is responsible for completion of cytosine methylation, thereby separating *NSUN2* from the modified cytosine site. We focused on modification of the C271 site of *NSUN2* with varied lysines using genetic encoding expansion and, subsequently, evaluated the effect of *NSUN2* C271 mutations on its activity and gene upregulation. Lysines with four different modifications were successfully installed at the C271 site of *NSUN2*. Reports indicate that *CDK1* is an upregulated gene of *NSUN2* [28,29,30]. *NSUN2* is highly expressed in various tumor cells, and expression of *NSUN2* promotes the translation process of *CDK1*, promoting cell cycle and proliferation [30,31,32]. Furthermore, RT-PCR and cell proliferation assays revealed that the C271 mutation had a minimal influence on mRNA expression levels of upregulated factor *CDK1*, but significantly inhibited *CDK1* functions. Moreover, C271 mutations with S1–S4 inhibited HeLa cell proliferation. We postulate that C271 site mutation can inhibit the separation of *NSUN2* from the target RNA sequence after completing the catalysis of m^5^C, thereby affecting RNA functions, including the translation process of the target protein, *CDK1* [28] to inhibit cell proliferation.

First, natural lysines modified with special groups were synthesized by organic synthesis. Second, through two rounds of positive screening and one round of negative screening, we evaluated the *MbPylRS*-tRNA_CUA_ unnatural lysine substitution system, which specifically recognizes lysines with a specific group. Non-natural lysine substitution at C271 active site of *NSUN2* and the subsequent fluorescent labeling were realized through the click reaction. Furthermore, we investigated the function of *NSUN2* with mutants (S1–S4), its upregulated *CDK1* gene, and its effect on cell proliferation. In summary, through genetic encoding expansion, we constructed the *NSUN2* model, whose important sites were mutated with unnatural lysine bearing N_3_, PBin, NO_2_, and CF_3_, among others. Moreover, through the click reaction, efficient labeling, and regulation of *NSUN2* was verified, laying the basis for further studies on the function and regulatory mechanism of upregulated genes.

## Supplementary Materials

The following are available online at https://www.mdpi.com/article/10.3390/genes12101488/s1, *m^5^C*, 5-methylcytosine; *DNMT2*, DNA methyltransferase; *ALYREF*, Aly/REF export factor; *PPIs*, protein–protein interactions; *PBBK*, 2-Amino-6-[4-(4,4,5,5-tetramethyl-[1,3,2]-dioxa-borolan-2-yl)-benzyloxy-carbonyl amino]-hexanoic acid; THF, tetrahydrofuran; r.t., room temperature; Boc-l-lysine, Nalpha-(tert-butoxycarbonyl)-l-lysine; HRMS, high-resolution mass spectrometer; EGFR, enhanced green fluorescent protein; *DsRed*, a mutant form of DsRed from Discosoma sp.; TCEP, tri(2-carboxyethylphosphine hydrochloride; *CDK*, cyclin-dependent kinases; *NSUN2*, *NOP2/SUN* RNA methyltransferase 2; DBCO-Cy5, DBCO-Sulfo-Cy5; *FTO*, fat mass and obesity; *KRAS*, Kirsten rat sarcoma viral oncogene homolog. Figure S1. 1H-NMR spectrum of Boc-S1; Figure S2. 13C-NMR spectrum of Boc-S1; Figure S3. HR-MS spectrum of Boc-S1; Figure S4. 1H-NMR spectrum of S1; Figure S5. 13C-NMR spectrum of S1; Figure S6. HR-MS spectrum of S1; Figure S7. 1H-NMR spectrum of S2; Figure S8. 1H-NMR spectrum of Boc-S3; Figure S9. 13C-NMR spectrum of Boc-S3; Figure S10. 1H-NMR spectrum of S3; Figure S11. 13C-NMR spectrum of S3; Figure S12. 1H-NMR spectrum of S4; Figure S13. 13C-NMR spectrum of S4; Figure S14. HR-MS spectrum of compound S4; Figure S15. Proton NMR results of S3 with TCEP; Figure S16. Crystal structure analysis of typical NSUN2 with ligand SAM; Figure S17. Overall illustration of screen of systems for non-canonical amino acids (S1–S4); Figure S18. Proton NMR of DBCO-Cy5 dye; Figure S19. Detection of specific sites of unnatural and unnatural amino acid insertion proteins by dual fluorescence reporting system. Scale bar: 50 μM; Figure S20. (A) Western blot the effect of unnatural amino acid substitution on the expression of NSUN2 protein was detected. (B) The blast results of the gene mutation of MbPylRS which have been screened specific to S1-S4, respectively. P.C. represents positive control assay; Figure S21. (A) Detection of NSUN2 labeling by S3 (Lys-N3) in eukaryotic cells by dual fluorescent reporter gene. Scale bar: 50 μM. (B) Detection of the read though of UAA with or without lysine analogs. The results demonstrated that the screened MbPylRS-tRNA system was used specifically to the target lysine analogs, such as S1 in Figure S21B. However, there is little read though of UAA without lysine analogs; Figure S22. Detection of transcription by qPCR. O.P. represents overexpression. K.O. means knockout; Figure S23. The knockout results of NSUN2 in the knockout cell line. N.C. represents negative control; Scheme S1. Synthesis of compound S1; Scheme S2. Synthetic route for compound S2; Scheme S3. Synthetic routes for compound S3; Scheme S4. Synthetic routes for compound S4; Scheme S5. Illustration of labeling of azido-containing amino acid using DBCO-Cy5; Scheme S6. Overall Illustration of labeling of azido-containing NSUN2 using DBCO-Cy5. Table S1: Calculated conversion of the reaction of S3 with TCEP. Chart S1: Reaction plot of S3 with TCEP.
