# Supplementary material for: Regulation and Site-Specific Covalent Labeling of NSUN2 via Genetic Encoding Expansion
Source: Genes (Basel). 2021 Sep 24;12(10):1488. doi: 10.3390/genes12101488 (PMC8535899; doi:10.3390/genes12101488)
Supplement: Supplementary file 1 [file genes-12-01488-s001.zip › genes-1333034-supplementary.pdf]

Supporting Information

Regulation and Site-specific Covalent Labeling of *NSUN2* Enable by Genetic Encoding Expansion

Jizhong Zhao,†, [a] Hongmei Hu,†, [a] Sheng Wang [a] and Rui Wang*,[a]

[a]Hubei Key Laboratory of Natural Medicinal Chemistry and Resource Evaluation, School of Pharmacy, Tongji Medical College, Huazhong University of Science and Technology, Wuhan, Hubei 430030, People's Republic of China.

Corresponding authors: ez_rwang@hust.edu.cn.

Table of Contents

1. Materials.......Page2
2. Synthetic approaches for unnatural lysines (S1-S4) Page3
3. Dynamic property of 6-azido lysine (S3) with TCEP.......Page13
4. Sequence of *NSUN2* and crystal structure analysis of *NSUN*.......Page15
5. Screening strategy for the site-specific incorporation of UAA.......Page16
6. Labeling strategy of 6-azido site-specific *NSUN2*.......Page18
7. Overall protocol for unnatural lysine substitution.......Page20
8. Bio-orthogonal reaction to label *NSUN2* and detect its subcellular localization.......Page23
9. Effect of *NSUN2* active site mutation on function.......Page24
10. The effect of substitution of *NSUN2* with unnatural lysine on function.......Page26
11. References.......Page27

MATERIALS

All chemicals were purchased from commercially available sources otherwise stated including *Innochem* (Beijing, China), *Aladdin* (Shanghai), *Ark* (Shanghai), *TCI* (Shanghai), *Sigma-Aldrich Inc*. (Shanghai). All solvents were used directly purchased from *Innochem* (Shanghai) without further purification. Buffers including phosphate buffered saline (PBS) and Tris(hydroxymethyl) aminomethane (Tris) were purchased from *Innochem* (Beijing). 4-(2-Hydroxyethyl)piperazine-1-ethanesulfonic acid (HEPES, Cas # 7365-45-9), acrylamide and thiazolyl blue tetrazolium bromide (MTT, Cas # 298-93-1) were purchased from *Sigma-Aldrich* (Shanghai).

NMR were done on a *Bruker AM-400 spectrometer*. Mass spectra was performed on *Advion Expression L* (Bohui Innovation Biotechnology) using electrospray ionization (ESI). UV spectra was performed on *Perkin Elmer Lambda 365* (German). Gel Imaging was performed using *Pharos FX Molecular Imager* (Bio-Rad, USA). Confocal microscope images were analyzed on *Zeiss LSM780*. Flow cytometry were analyzed on an *LSR-II Flow Cytometer* (BD Biosciences) and data were analyzed using *FlowJo* software (Tree Star).

SYNTHETIC APPROACHES FOR UNNATURAL LYSINES (S1-S4).

(1) Synthesis of substrate **S1**.[1]

Ref 1. Chen, P. R., Groff, D., Guo, J., Ou, W., Cellitti, S., Geierstanger, B. H., Schultz, P.G. *Angew. Chem. Int. Ed. Engl*. **2009**, *48* (22), 4052-5.

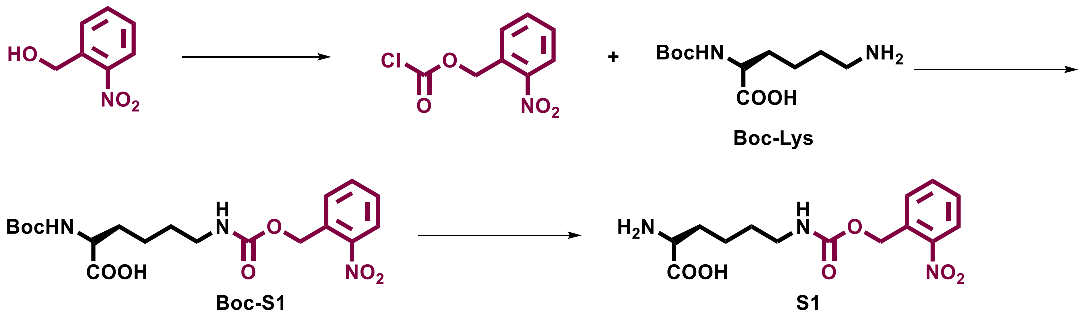

Scheme S1. Synthesis of compound **S1**.

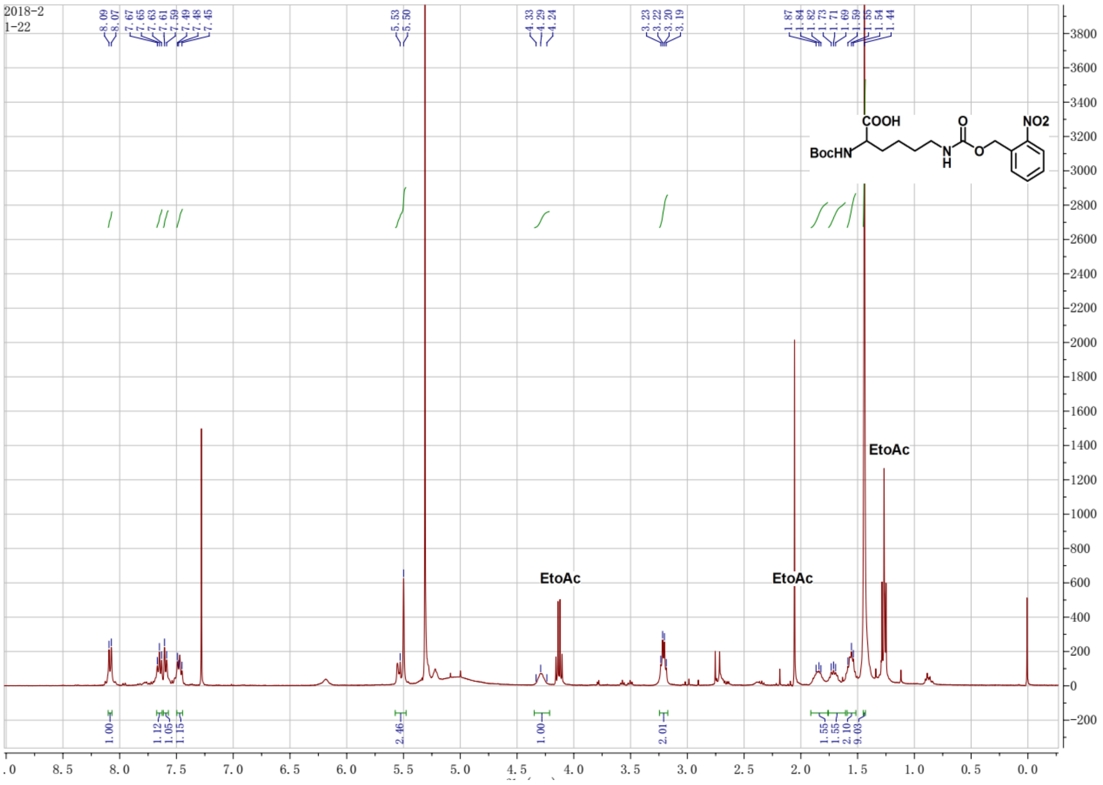

Figure S1. ^{1}H-NMR spectrum of Boc-**S1.**

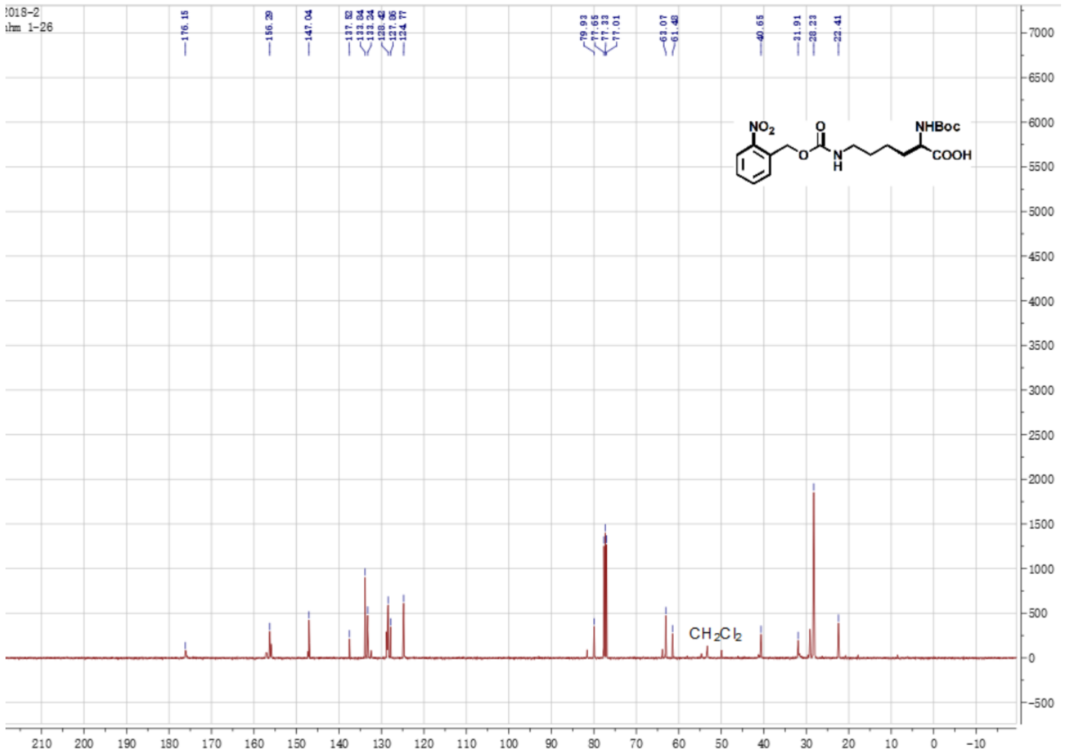

Figure S2. ^{13}C-NMR spectrum of Boc-**S1.**

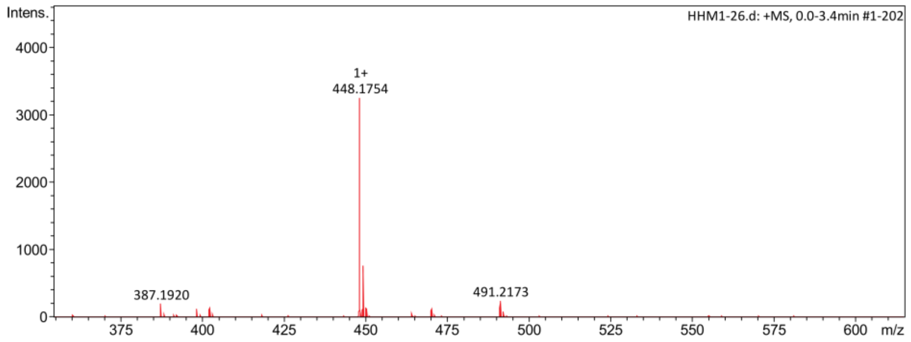

Figure S3. HR-MS spectrum of Boc-**S1.**

Step3: Boc-**S1** (1.2 g, 2.8 mmol) was dissolved in CH_2Cl_2 (10.0 mL) followed by TFA (10.0 mL) at 0°C under N_2 atmosphere. The reaction mixture was stirred for 0.5h at r.t. Et_2O (30.0 mL) was added at 0°C, the **S1** (1.1 g) was precipitated out and was obtained in 93% yield. **^{1}H NMR** (400 MHz, D_2O) δ 8.22 (d, *J* = 8.0 Hz, 1H), 7.84 (t, *J* = 8.0 Hz, 1H), 7.72-7.66 (m, 2H), 5.53 (s, 2H), 3.87-3.83 (m, 1H), 3.31-3.21 (m, 2H), 1.99-1.92 (m, 2H), 1.64-1.59 (m, 2H), 1.51-1.47 (m, 2H); **^{13}C NMR** (101 MHz, D_2O) δ 172.24, 157.92, 146.83, 134.42, 132.29, 129.01, 128.70, 125.02, 66.55, 63.56, 52.90, 39.91, 29.41, 21.44. **MS (ESI)**: $[M-H]^{-}$ = 323.8; **HRMS**: Calcd for $C_{14}H_{19}N_3O_6$ $[M+ CH_3CN+ MeOH]^{+}$ = 398.1802; Found: 398.2461.

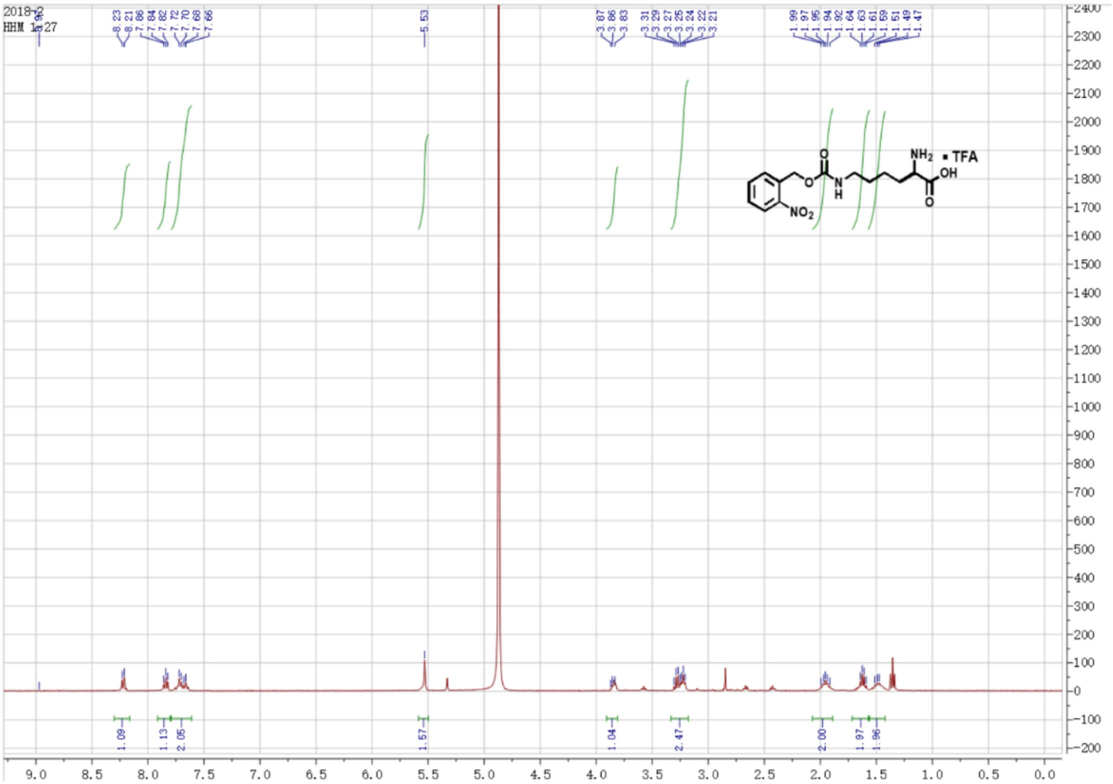

Figure S4. ^{1}H-NMR spectrum of **S1.**

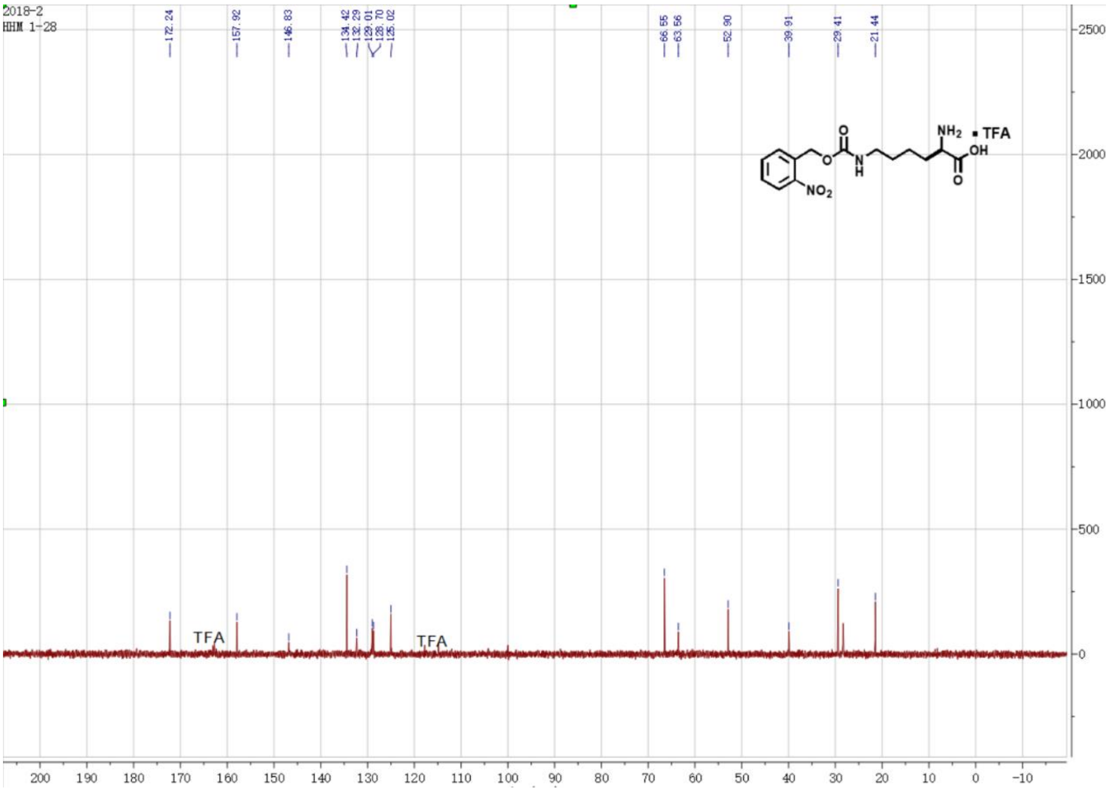

Figure S5. ^{13}C-NMR spectrum of **S1.**

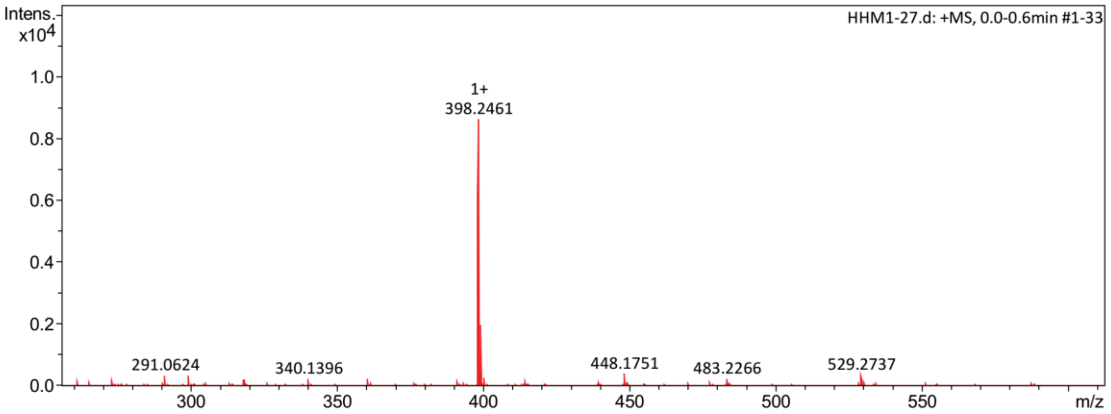

Figure S6. HR-MS spectrum of **S1.**

(2) Synthesis of substrate **S2**.

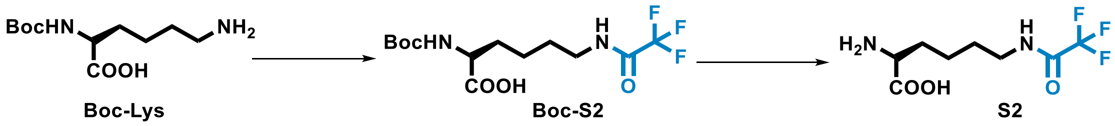

Scheme S2. Synthetic route for compound **S2**.

Step1: To a stirred solution of Boc-*L*-lysine (760 mg, 3.1 mmol) in anhydrous pyridine (10 mL) was added trifluoroacetic anhydride (479 μL, 3.4 mmol) at 0°C under N_2 atmosphere. The mixture was slowly warmed to room temperature and continue to stir for 24 hours. Solvents were removed under reduced pressure and the crude product was purified by silica-gel chromatography to give Boc-**S2** as yellowish solid directly for next step without further purification.

Step2: Boc-**S2** obtained above was dissolved in ether (6.0 mL) at 0°C under N_2 atmosphere. HCl-dioxane (1.0 mL, 4.0 M) was added and slowly warmed to room temperature. Solvents were removed and precipitated by ether to afford **S2** (748 mg) in quantitative yield. ^{1}H-NMR (400 MHz, $CDCl_3$) δ 3.30-3.27 (m, 2H), 3.26-3.20 (m, 1H), 1.99-1.92 (m, 2H), 1.64-1.59 (m, 2H), 1.51-1.47 (m, 2H); Advion MS calculated for $[M+H]^+$ = 242.1; observed 242.1.

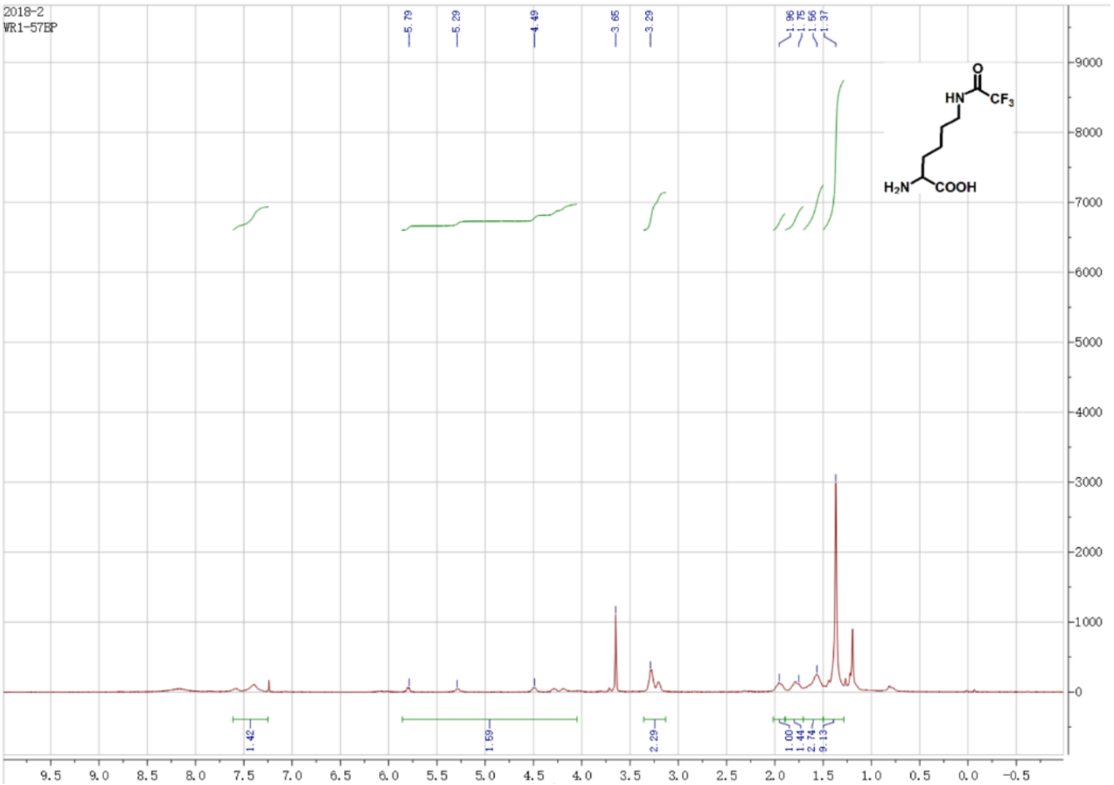

Figure S7. ^{1}H-NMR spectrum of **S2.**

(3) Synthesis of substrate **S3**.[2]

Ref. 2. Wang, Z. A., Kurra, Y., Wang, X., Zeng, Y., Lee, Y. J., Sharma, V., Lin, H., Dai, S. Y., Liu, W. R. *Angew. Chem. Int. Ed. Engl.,* **2017**, *56* (6), 1643-1647.

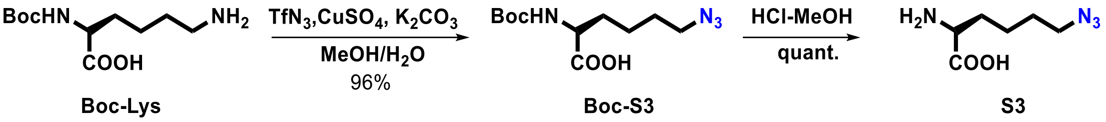

Scheme S3. Synthetic routes for compound **S3**.

Step1: Triflic azide preparation: sodium azide (5.5 mmol) was dissolved in a mixture of H_2O (15 mL) and CH_2Cl_2 (10 mL). Trifluoromethanesulfonic anhydride (0.2 mL) was slowly added at 0°C. Two hours later, the aqueous phase was extracted with CH_2Cl_2 (7.5 mL × 2). The combined organic phase was washed with saturated Na_2CO_3. The triflic azide prepared was used directly for next step reaction without further purification.

Boc-*L*-lysine (0.6 mmol, 150 mg) was suspended in a mixture of MeOH (6.0 mL) and H_2O (2.0 mL), $CuSO_4$•$5H_2O$ (30 mg) was added in one portion. K_2CO_3 (100 mg) was added to the mixture to adjust the pH = 9.5. The triflic azide

solution obtained above was added to the mixture, and continue to stir for overnight at room temperature. CH_2Cl_2 (150 mL) was added to enhance phase separation. The organic phase was extracted with H_2O (50 mL × 4). The combined aqueous phase was acidified to pH = 2 with hydrochloric acid. The solution was then extracted with CH_2Cl_2 (50 mL × 6). The extract was dried over Na_2SO_4 and concentrated under reduced pressure. The resulting azide Boc-**S3** (160 mg, 98% yield) was obtained (TLC, CH_2Cl_2: MeOH: AcOH = 10: 1: 0.1). **^{1}H NMR** (400 MHz, $CDCl_3$) δ 8.82 (s, 1H, COOH), 6.63 - 5.22 (m, 1H, CH), 4.24 (d, *J* = 80 Hz, 1H, 1H of CH_2), 3.29 (t, *J* = 12 Hz, 2H, CH_2), 1.89 (s, 1H, 1H of CH_2), 1.67 (d, *J* = 36 Hz, 3H, CH & CH_2), 1.45 (s, 9H, CH_3 × 3). **^{13}C NMR** (100 MHz, $CDCl_3$) δ 155.74, 81.97, 80.40, 51.11, 31.93, 28.40, 28.27, 22.55. **MS (ESI)**: Calculated for $[M-H]^-$ = 271.1; Observed 271.2.

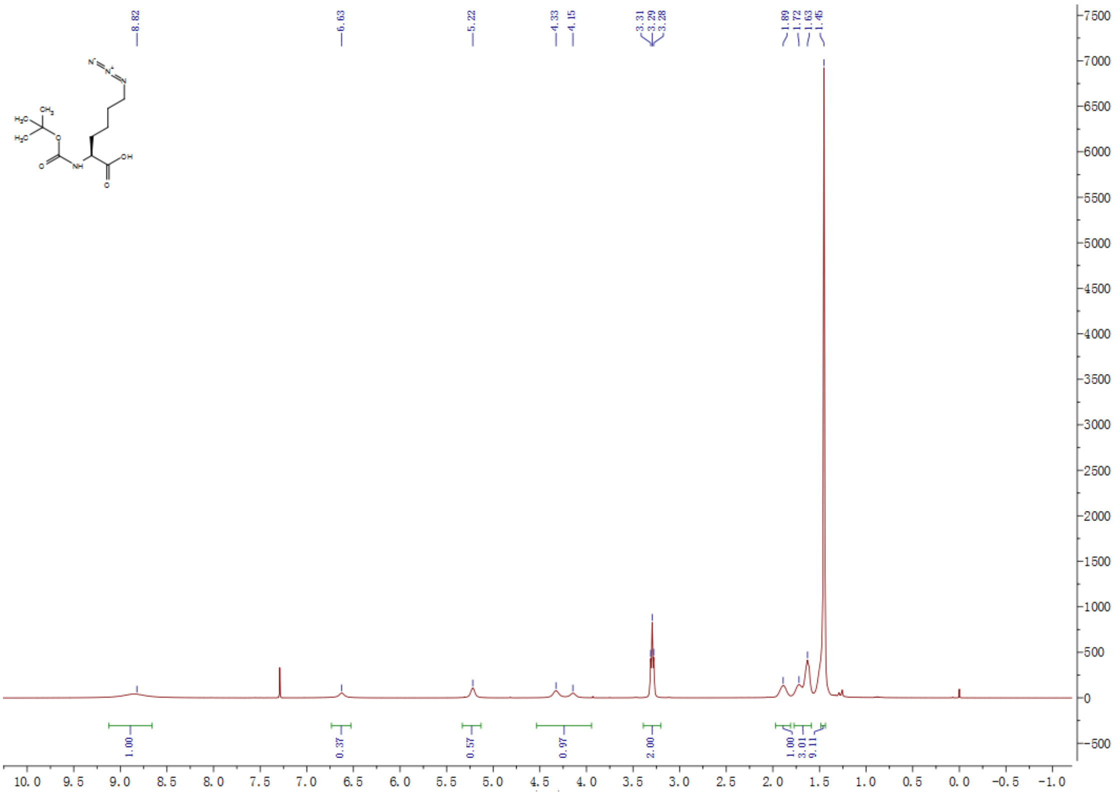

Figure S8. ^{1}H-NMR spectrum of Boc-**S3.**

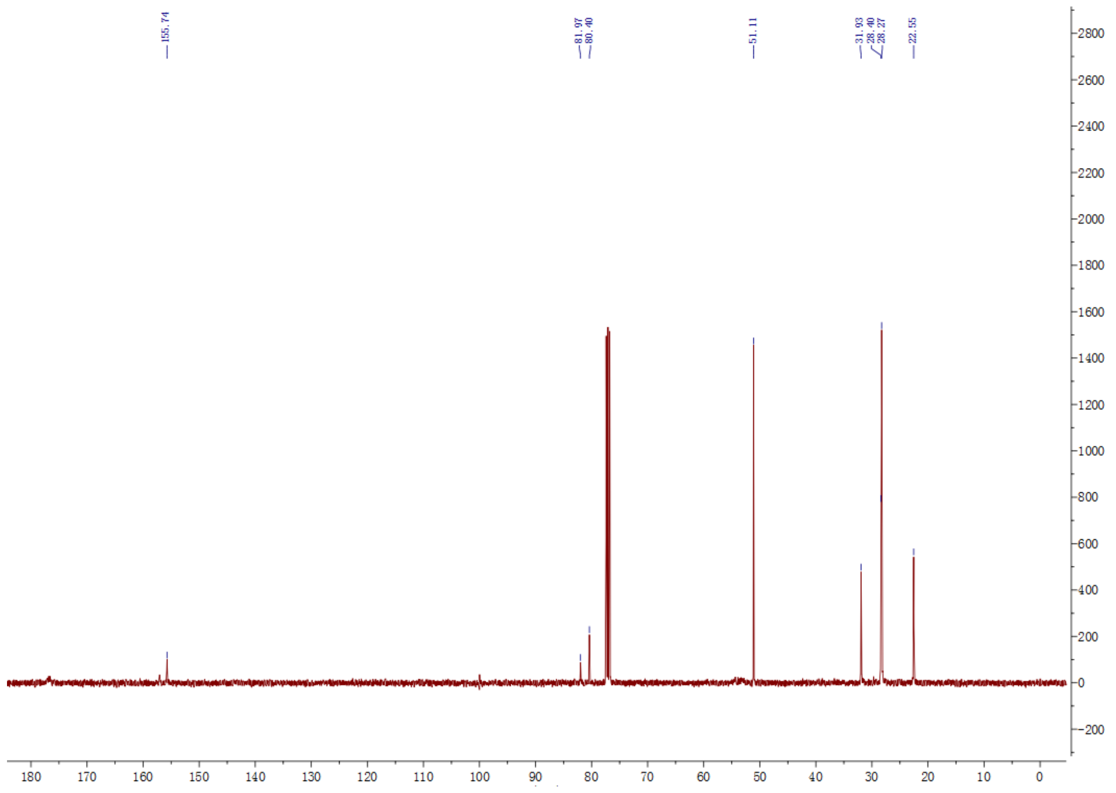

Figure S9. ^{13}C-NMR spectrum of Boc-**S3.**

Step2: Boc-Anl-OH (160 mg, 0.58 mmol) was dissolved in 4.0 N HCl in MeOH (5.0 mL) under ice-water bath. After stirring at room temperature for 4 hours, TLC analysis showed that the reaction was completed. The reaction mixture was concentrated under reduced pressure. The residues were dissolved in D. I. water and lyophilized to get a yellow solid **S3** (100 mg, 0.58 mmol, 100% yield). **^{1}H NMR** (400 MHz, D_2O) δ 4.10 (dt, *J* = 56 Hz, 1H, C<u>H</u>), 3.83 (s, 2H, N<u>H</u>2), 3.34 (t, *J* =12 Hz, 2H, C<u>H</u>2), 2.05 - 1.90 (m, *J* = 60 Hz, 2H, C<u>H</u>2), 1.63 (s, *J* = 24 Hz, 2H, C<u>H</u>2), 1.53 - 1.45 (m, *J* = 32 Hz, 2H, C<u>H</u>2). **^{13}C NMR** (100 MHz, D_2O) δ 172.23 (<u>C</u>OOH), 170.67 (<u>C</u>OOH), 53.61 (<u>C</u>H), 52.96 (<u>C</u>H), 52.78 (<u>C</u>H), 50.67 (<u>C</u>H2), 50.64 (<u>C</u>H2), 29.35 (<u>C</u>H2), 29.27 (<u>C</u>H2), 27.58 (<u>C</u>H2), 27.51 (<u>C</u>H2), 21.59 (<u>C</u>H2). **MS (ESI)**: Calculated for $[M+H]^+$ = 173.1; Observed 173.1.

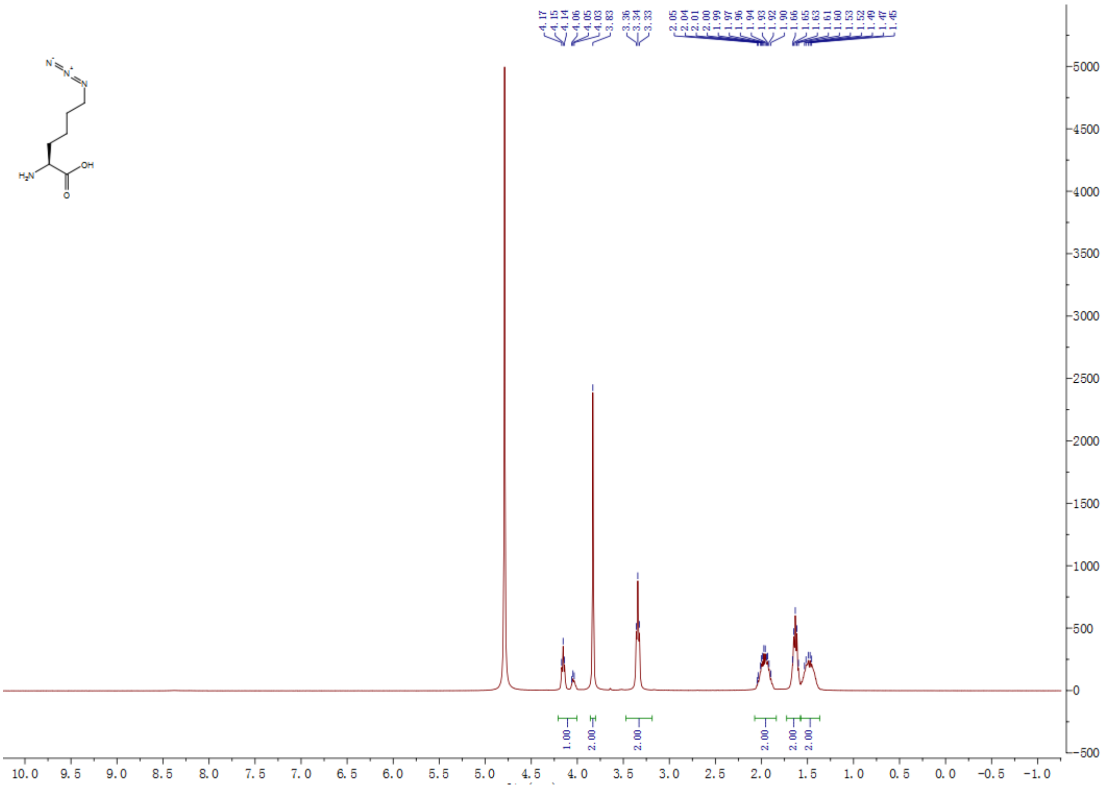

Figure S10. ^{1}H-NMR spectrum of **S3.**

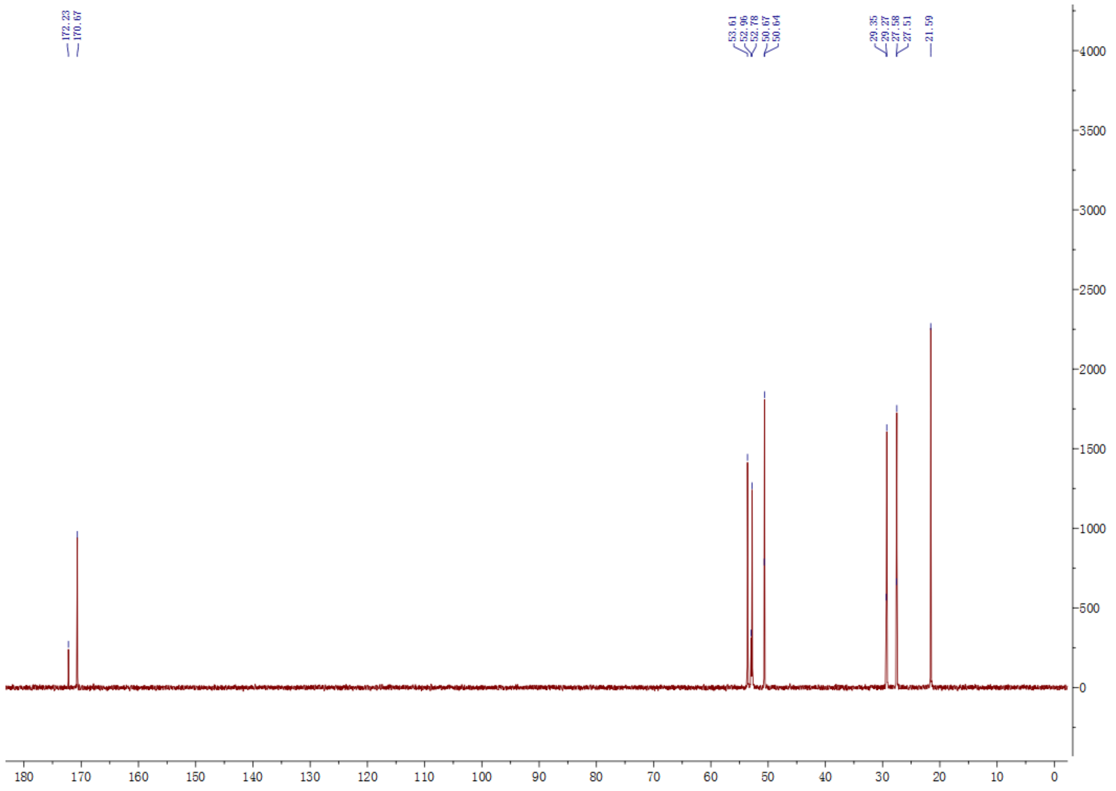

Figure S11. ^{13}C-NMR spectrum of **S3.**

(4) Synthesis of substrate **S4**.

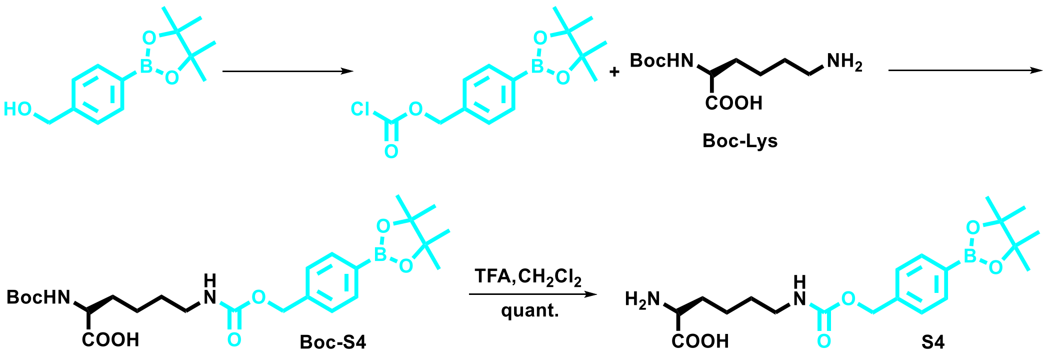

Scheme S4. Synthetic routes for compound **S4.**

S4 was obtained followed our unpublished results.[3] *Ref. 3*. Also see main context reference 23. **^{1}H NMR** (400 MHz, D_2O) δ 7.70 (d, J = 7.9 Hz, 1H), 7.34 (d, J = 7.8 Hz, 1H), 5.04 (s, 1H), 3.81 (t, J = 6.0 Hz, 1H), 3.05 (t, J = 6.2 Hz, 1H), 1.82 (t, J = 16.9 Hz, 1H), 1.63- 1.38 (m, 1H), 1.40-1.24 (m, 1H), 1.13 (s, 6H). **^{13}C NMR** (101 MHz, D_2O) δ 172.17, 162.59 (q, J = 35.9 Hz), 158.02, 134.62, 134.28 (d, J = 69.0 Hz), 126.71 (s), 116.31 (q, J = 292.4 Hz), 84.17, 75.53, 66.54, 52.79, 39.85, 29.31, 28.38, 24.07, 23.88 (d, J = 34.6 Hz), 21.43. **MS (ESI)**: $[M+H]^+$ = 407.6. **HRMS (MS)**: Calcu. for $C_{20}H_{31}BN_2O_6$ $[M+ CH_3CN+ H]^+$: 448.2619; Found: 448.1744.

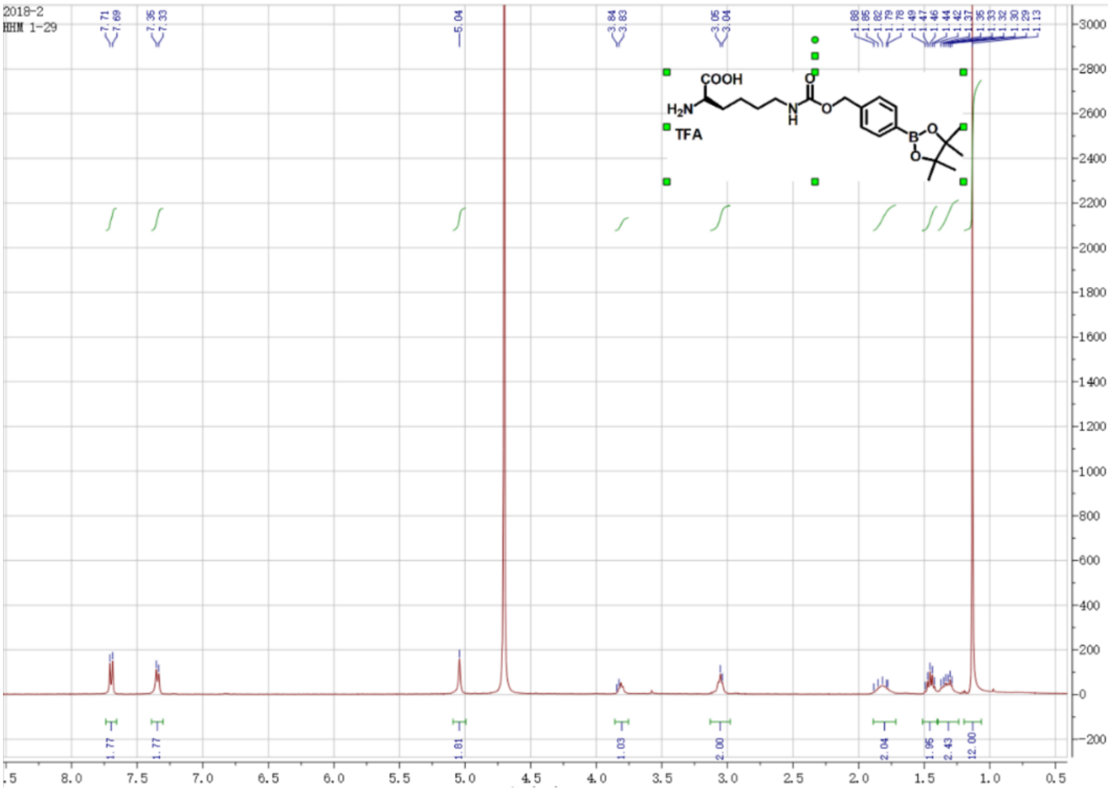

Figure S12. ^{1}H-NMR spectrum of **S4.**

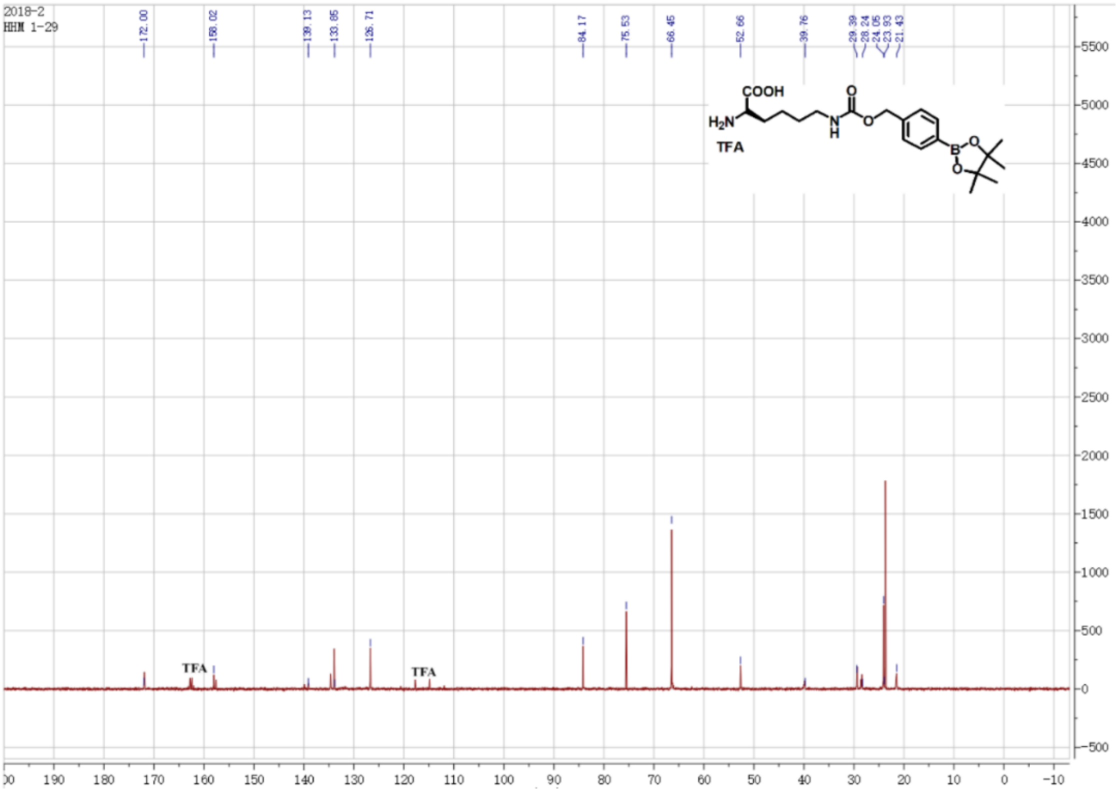

Figure S13. ^{13}C-NMR spectrum of **S4.**

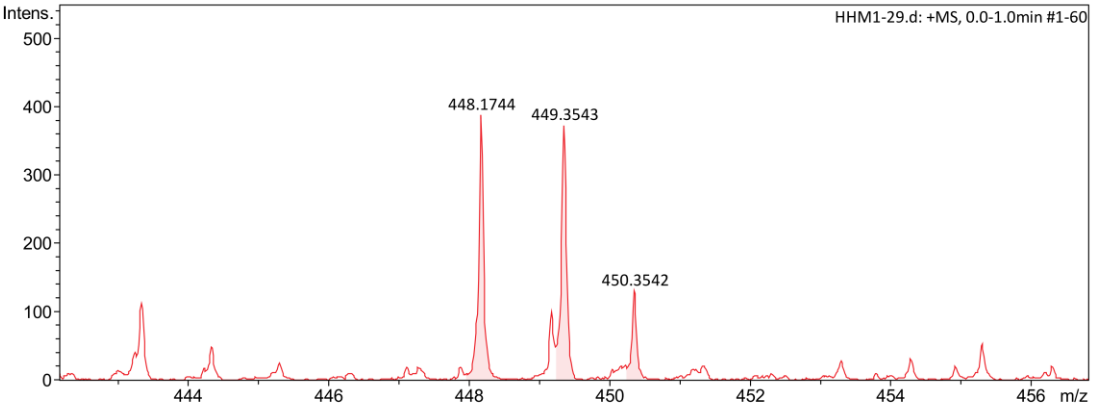

Figure S14. HR-MS spectrum of compound **S4.**

DYNAMIC PROPERTY EVALUATION OF 6-AZIDO LYSINE (S3) WITH TCEP

The ^{1}H NMR spectra were recorded on a 400 MHz Bruker NMR machine. For reactions with TCEP, Lys-N_3 (**S3**, 0.01 mmol) were dissolved in D_2O (0.5 mL, final concentration = 20 mM). TCEP (0.1 mmol) was then added to the solution (final concentration = 200 mM), and the reaction mixture was allowed to stand at room temperature. At predetermined time intervals, the solution mixture was taken for ^{1}H-NMR analysis as previously described.

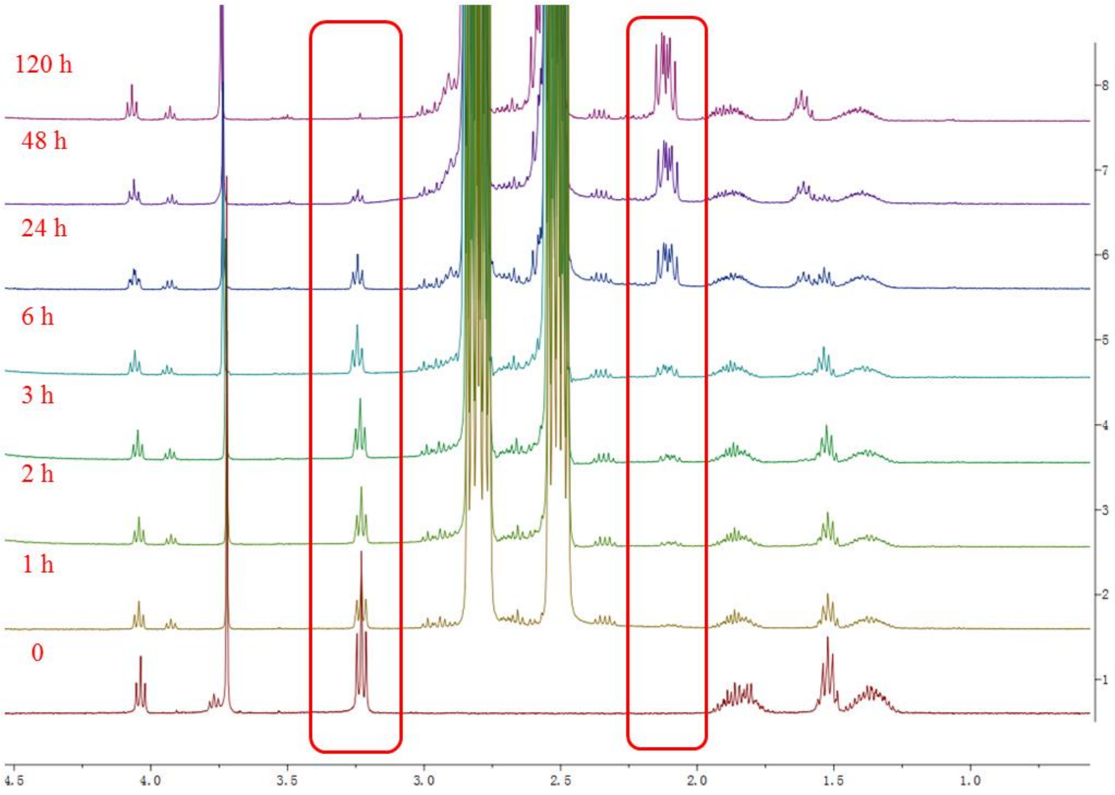

Figure S15. Proton NMR results of **S3** with TCEP.

Entries	Starting Material (%)	Product (%)
0	100	0
1h	85.0	15
2h	82.0	18
3h	79.0	21
6h	73.0	27
24h	43.0	57
48h	23.0	77
120h	3.0	97

Table S1. Calculated conversion of the reaction of **S3** with TCEP.

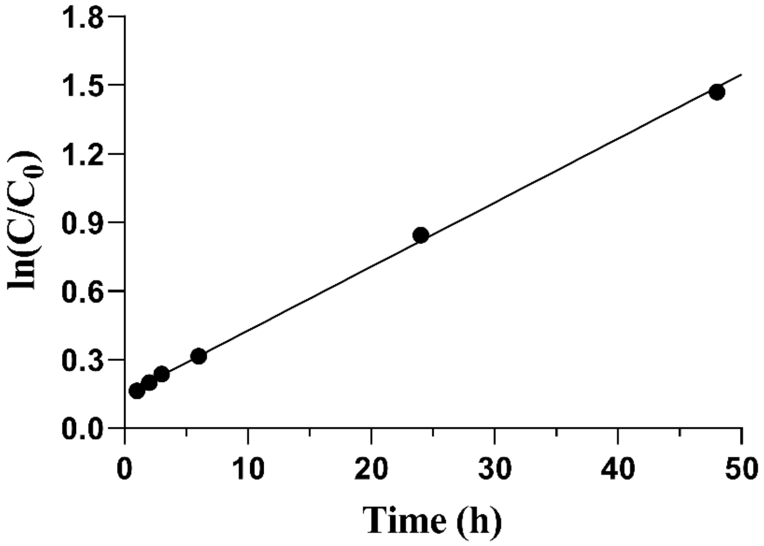

Chart S1. Reaction plot of **S3** with TCEP.

Representative data for rate determination of Lys-N_3 conversion under pseudo first-order conditions. Y = **<u>0.02798</u>***X + 0.1462, R^2 = 0.9999.

SEQUENCE OF *NSUN2* AND CRYSTAL STRUCTURE ANALYSIS OF ANALYSIS OF *NSUN4*.

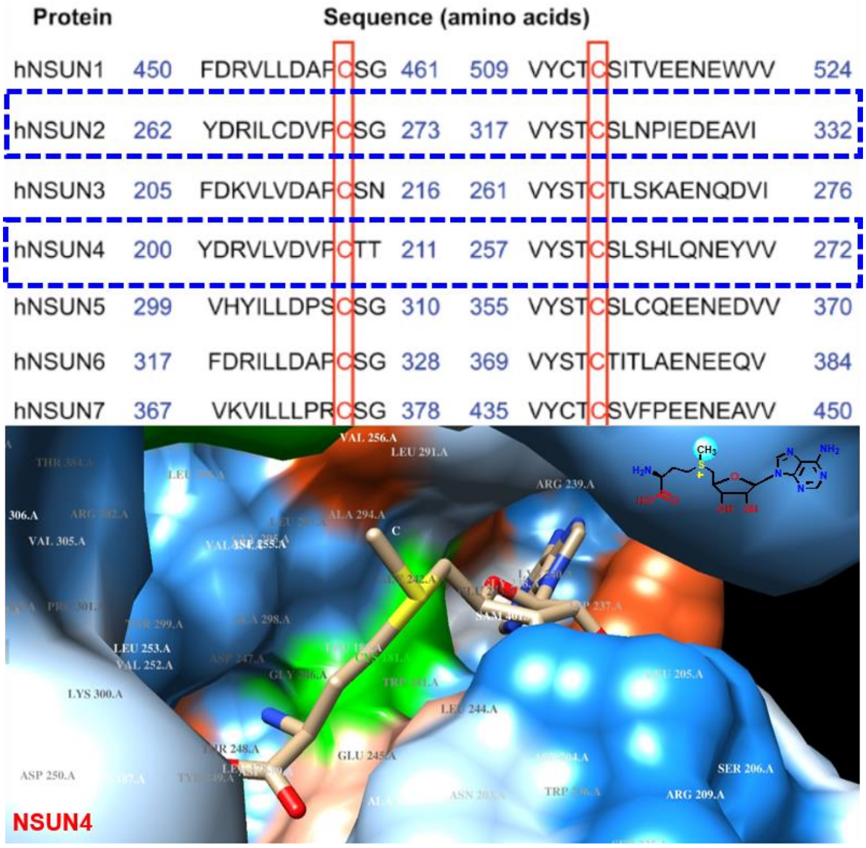

Figure S16. Crystal structure analysis of typical *NSUN2* with ligand SAM.

SCREENING STRATEGY FOR THE SITE-SPECIFIC INCORPORATION OF UAA.

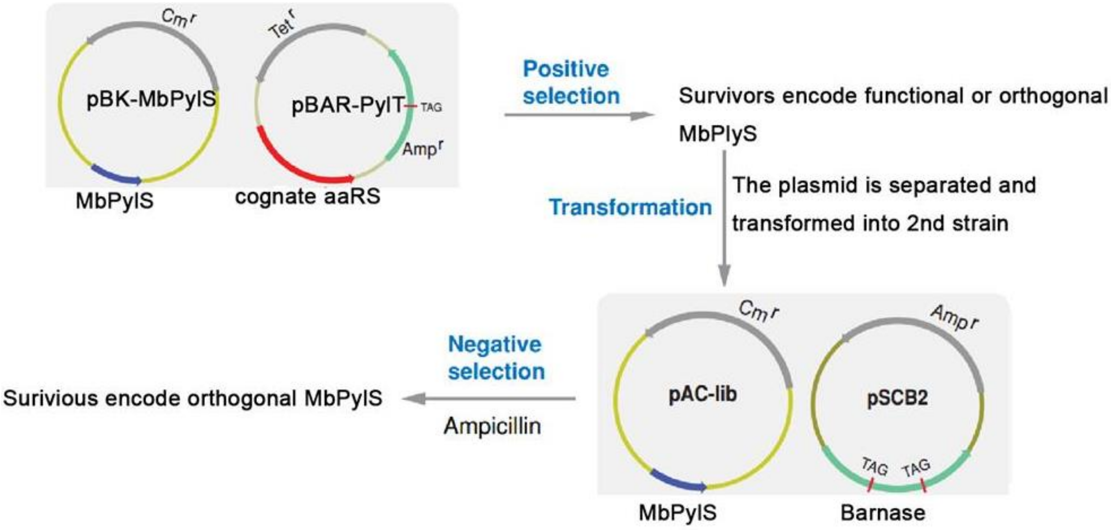

Figure S17. Overall illustration of screen of systems for non-canonical amino acids **(S1-S4).**

From this mutant library, *MbPylRS* mutants that can specifically recognize lysine derivatives were selected in three steps.

The first step is a positive screening, in which two sites in the tetracycline gene sequence are replaced with TAG amber codons, and in the presence of the *MbPylRS*-tRNA$_{CUA}$ system, if the *MbPylRS* mutant can specifically recognize the band Lysine derivatives with specific modification groups can convert the stop codon into an intentional codon that can encode lysine derivatives. Thus, tetracycline can be expressed smoothly. In the medium containing tetracycline (25 µg/ml), the strain that can encode TAG can survive, otherwise it cannot survive. In this step, all the survival clones are collected about 10*^{7} clones.

The second screening step is a negative screening system. In this system, Barnase ribonuclease is selected. Because Barnase has strong cytotoxicity, cells cannot survive in strains expressing Barnase. Here, the two sites in Barnase were also mutated to amber codons. On the basis of positive

screening, negative screening was performed here. The culture medium contained no specific lysine derivatives and contained *MbPylRS*-$tRNA_{CUA}$ system and Barnase negative screening system strains, if the strains can survive, it indicates that *MbPylRS*-$tRNA_{CUA}$ has specific recognition for specific lysine derivatives. However, if the strain is unable to survive, it indicates that Barnase is expressed, which means that *MbPylRS*-$tRNA_{CUA}$ has no specific or poor specificity for the recognition of specific lysines, and can also recognize other amino acids. In this step about 10^{*4} clones were collected to be used for the next round selection.

The third round screening was same as the first round postive selection. The clones which have been selected though the above two screening will be further selected in tetracycline (50ug/ml) containing solid medium. In this step, about 100 clones were selected for each Lys derivatives. Then these clones will be cultured in LB medium with an increasing concentration of tetracycline (from 50 to 150 ug/ml). Finally, three clones were selected and prepared to be used for the UAA mutation proteins expression.

Through the above three step screening, the *MbPylRS*-$tRNA_{CUA}$ system with specific recognition and coding capabilities for target amino acid derivatives can be screened.

LABELING STRATEGY OF 6-AZIDO SITE-SPECIFIC NSUN2.

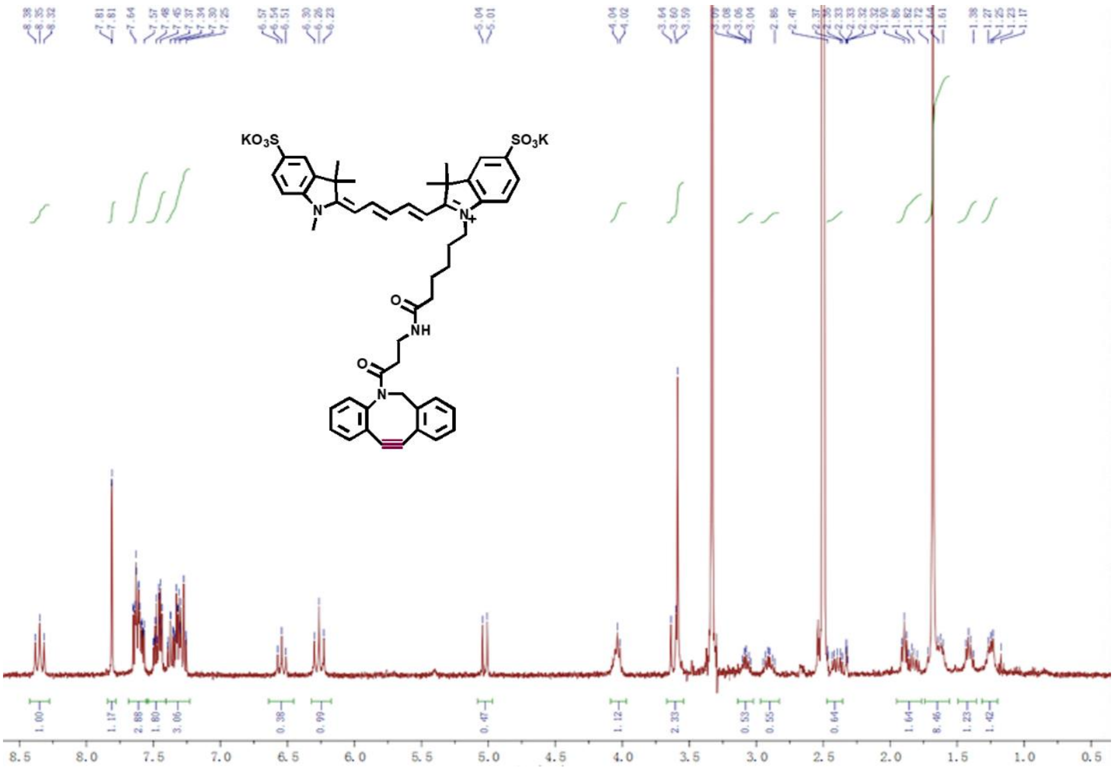

Figure S18. Proton NMR of DBCO-Cy5 dye.

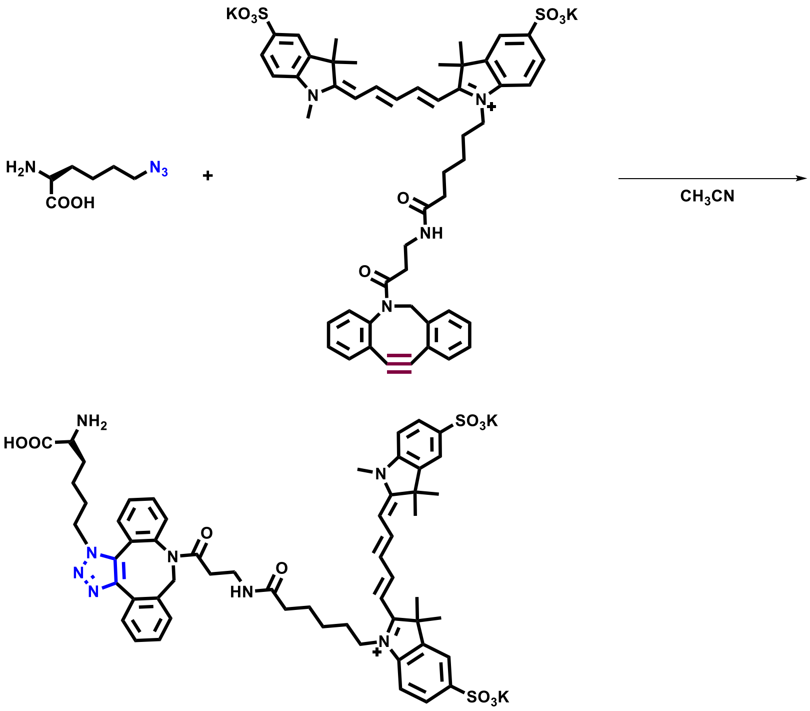

Scheme S5. Illustration of labeling of azido-containing amino acid using DBCO-Cy5.

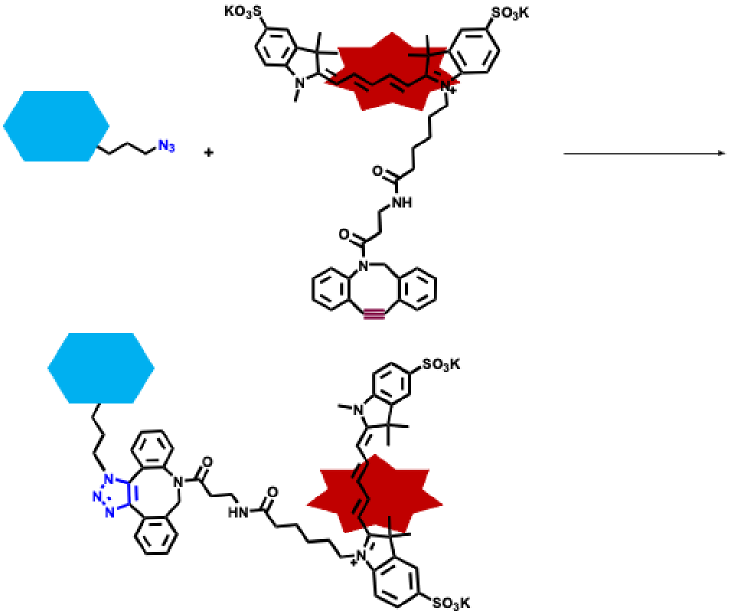

Scheme S6. Overall Illustration of labeling of azido-containing *NSUN2* using DBCO-Cy5.

OVERALL PROTOCOL FOR UNNATURAL LYSINE SUBSTITUTION.

Step 1.1 Screen specific MbPylRS.

Through the above two screening systems, after two rounds of positive screening and one round of invisible screening, the *MbPylRS*-tRNA$_{CUA}$ system with specific recognition and coding capabilities for target amino acid derivatives can be screened. Through this system, the *NSUN2* active site will be further realized. Point the replacement work of unnatural amino acids, as shown in **Figure S17.**

Step 1.2 Construction of a dual fluorescence reporter system for unnatural amino acid substitution detection.

The results of the experiment are shown in **Figure S19**, indicating that the expression of red fluorescent protein cannot be detected in the absence of **S1-S4**, and the expression of two fluorescent proteins can be detected in the presence of **S1-S4**, indicating that the above screening The *MbPylRS*-tRNA$_{CUA}$ can recognize specific unnatural amino acids and can encode amber codons.

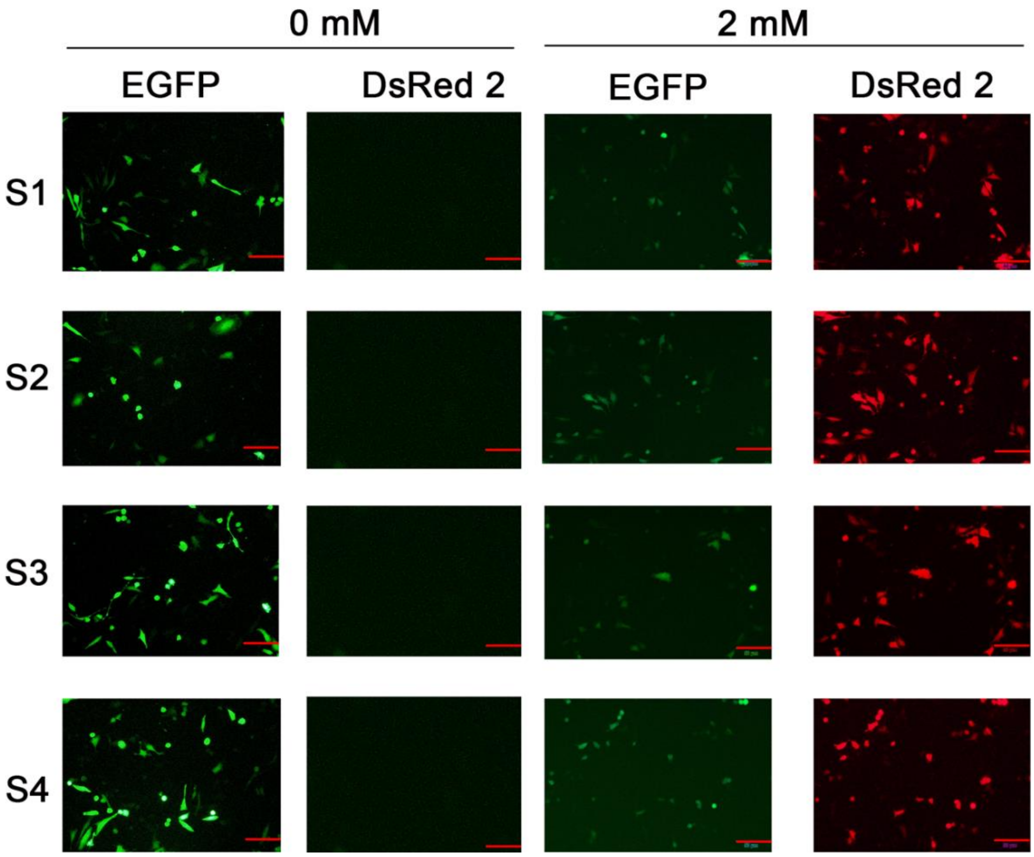

Figure S19. Detection of specific sites of unnatural and unnatural amino acid insertion proteins by dual fluorescence reporting system. Scale bar: 50 μM.

Simultaneous transfection of dual fluorescence reporter system and unnatural amino acid substitution system in HeLa cells. Then incubate for 24 hours in a DMEM medium without and with **S1-S4** (2.0 mM). Then the expression of fluorescent protein was detected under an inverted fluorescence microscope.

Step 1.3 Replacement of unnatural amino acids in the active site of NSUN2.

Through the above experiments, the construction and screening of the unnatural amino acid substitution system for the above four lysine derivatives in eukaryotic cells have been completed, and then the unnatural amino acid substitution of the active site C271 in *NSUN2* will be further implemented. C271 is an important active site of *NSUN2*, responsible for the separation of *NSUN2* from the substrate after catalysis.

Step 1.4 Western blot detection of the effect of unnatural amino acid substitution at C271 of NSUN2 on protein expression.

The expression level of *NSUN2* protein is relatively high in the presence of **S1** and **S3**, as shown in **Figure S20**.

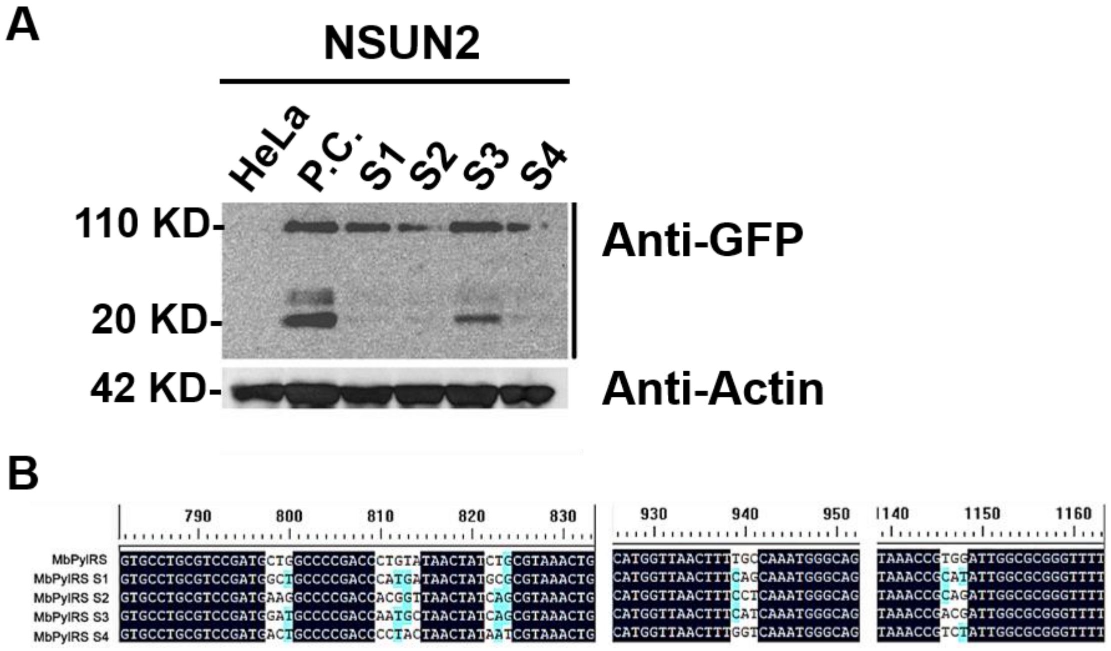

Figure S20. (A) Western blot the effect of unnatural amino acid substitution on the expression of *NSUN2* protein was detected. (B) The blast results of the gene mutation of *MbPylRS* which have been screened specific to **S1-S4**, respectively. P.C. represents positive control assay.

BIO-ORTHOGONAL REACTION O LABEL *NSUN2* AND DETECT ITS SUBCELLULAR LOCALIZATION.

The phenomenon of co-localization also shows that Lys-N_3 has achieved amino acid substitution at position C271 of *NSUN2*. This result also shows that this unnatural amino acid bio-orthogonal reaction can achieve efficient and stable protein labeling and tracing functions, as shown in **Figure S21**.

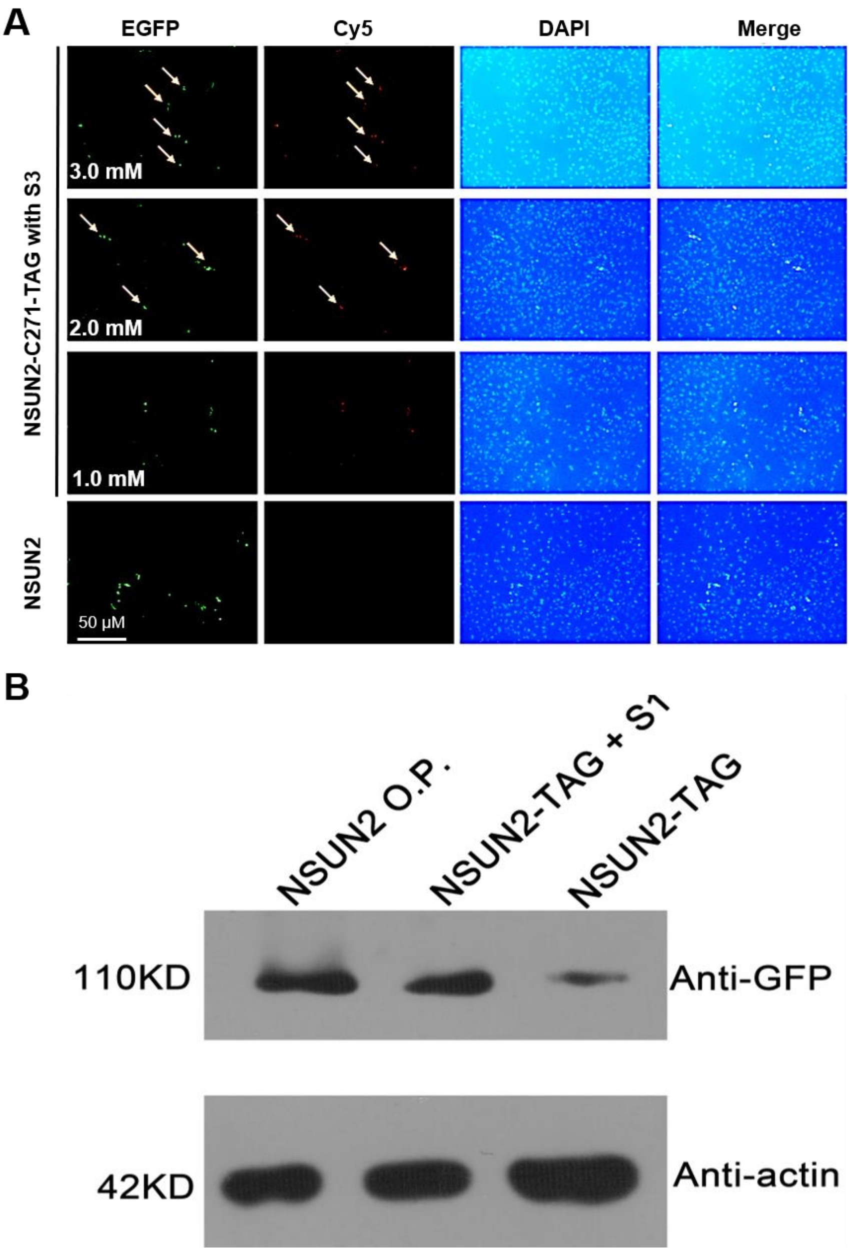

Figure S21. (A) Detection of *NSUN2* labeling by **S3** (Lys-N_3) in eukaryotic cells by dual fluorescent reporter gene. Scale bar: 50 μM. (B) Detection of the read though of UAA with or without lysine analogs. The results demonstrated that the screened *MbPylRS*-tRNA system was used specifically to the target lysine analogs, such as **S1** in **Fig. S21B**. However, there is little read though of UAA without lysine analogs.

EFFECT OF *NSUN2* ACTIVE SITE MUTATION ON FUNCTION

The above experiments have shown that the success of the *MbPylRS*-$tRNA_{CUA}$ unnatural amino acid replacement system, which the replacement of specific lysine derivatives at the C271 site of *NSUN2* in HeLa cells has been achieved. Next, further test the effect of *NSUN2* C271 site mutation on *NSUN2* activity has been evaluated.

First, the effect of mutation of *NSUN2* C271 to alanine on its functional activity was tested. As an RNA methyltransferase, *NSUN2* modifies RNA methylation and affects the function and stability of a variety of RNAs. Among them, *CDK1* has been found to be a downstream substrate of *NSUN2*. *NSUN2* is highly expressed in a variety of tumor cells, and the expression of *NSUN2* promotes the translation process of *CDK1*, thereby promoting the cell cycle process and promoting cell proliferation. Here, by knocking out the expression of *NSUN2* in HeLa cells and then filling the wild-type or C271 mutant of *NSUN2*, the proliferation ability of HeLa cells was tested by CCK8 kit, and the effect of *NSUN2* on *CDK1* transcription level was tested by RT-PCR. The experimental results showed that *NSUN2* had no significant effect on the transcription level of *CDK1*, as shown in **Figure 4**. However, after *NSUN2* was knocked out, the cell proliferation level was significantly reduced, and when the cells replenish wild-type *NSUN2*, the cell proliferation level returned to HeLa wild-type state. However, the cell proliferation level was significantly reduced after the mutation of *NSUN2* C271 site to alanine. It showed that the mutation of C271 site significantly inhibited the activity of *NSUN2*, as shown in **Figure 4**.

Figure 4. shows the effects of overexpression, knockout, and active site mutation (C271A) on the transcription level of the downstream gene *CDK1* in HeLa cells. The mRNA level of *CDK1* was detected by RT-PCR agarose gel electrophoresis. **Figure 4.** shows the effect of overexpression, knockout, and active site mutation (C271A) of *NSUN2* in HeLa cells on cell proliferation.

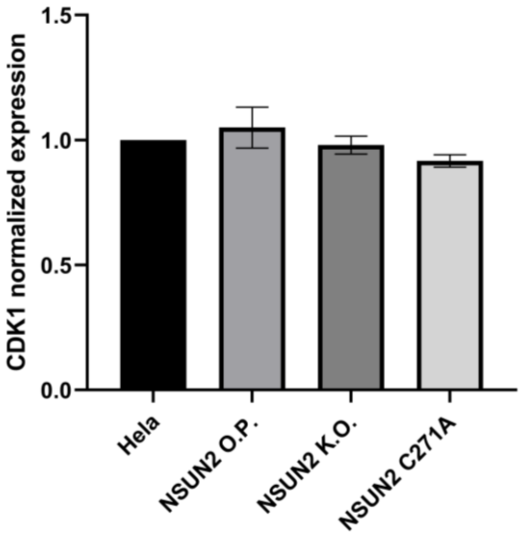

Figure S22. Detection of transcription by qPCR. O.P. represents overexpression. K.O. means knockout.

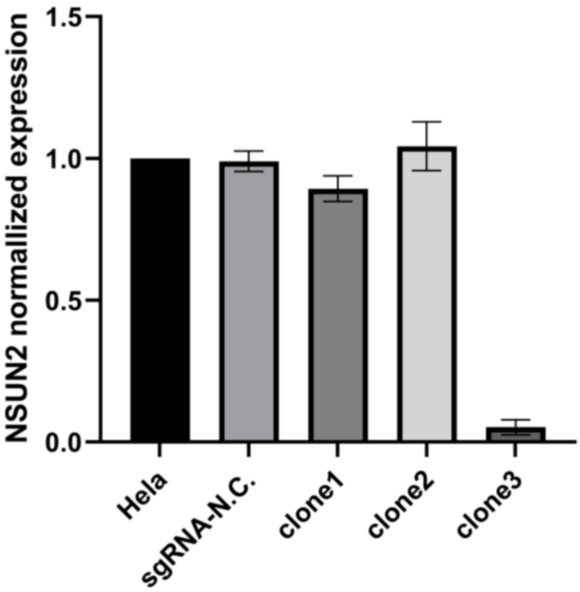

Figure S23. The knockout results of *NSUN2* in the knockout cell line. N.C. represents negative control.

Knockout assays. *NSUN2* was knockout by a CRISPR-Cas9 system. The sgRNA was designed to target the second exon of the gene sequence of *NSUN2*. The CRISPR-Cas9-NSUN2. K.O. plasmids were transfected into HeLa cells. Then the *NSUN2* knockout HeLa cells were selected in 96-well plates with G418 (500 ug/ml), and the monoclonal cells were detected by qPCR.

THE EFFECT OF SUBSTITUTION OF *NSUN2* WITH UNNATURAL LYSINE ON FUNCTION.

Next, the activity changes of *NSUN2* C271 active site after being replaced by unnatural amino acids were further examined. The *MbPylRS*-$tRNA_{CUA}$ unnatural amino acid substitution system and the *NSUN2* C271TAG mutant were co-expressed in HeLa cells, and the cells were cultured in a DMEM medium containing **S1-S4**, and then the proliferation ability of tumor cells was tested. The experimental results showed that the cell proliferation level in **S1** and **S3** medium is significantly lower than that of the wild type, and at the same time compared to **S2** and **S4**. This may be because **S1** and **S3** were more efficient than **S2** and **S4** for *NSUN2* active site amino acid substitution, as shown in **Figure 4D**.

After detecting the replacement of *NSUN2* C271 sites with **S1-S4** in HeLa cells, the changes in cell proliferation level were detected by CCK8.

In this part, lysines with specific modifications were synthesized by organic chemical synthesis, and through two rounds of positive screening and one round of negative screening, the lysines with specific groups could be specifically identified by use of *MbPylRS*-$tRNA_{CUA}$ unnatural amino acid substitution system. Upon establishment of the substitution of unnatural amino acids at C271, the active site of *NSUN2*, fluorescent labeling, the function of *NSUN2* and its downstream proteins were evaluated through studies of the effect on cell proliferation.

REFERENCES

K. Lang, L. Davis, A. Deiters, J. W. Chin. *Genetically encoded norbornene directs site-specific cellular protein labelling via a rapid bioorthogonal cycloaddition. Nat. Chem. 4*, 298-304 **(2012).**

J. Li, J. Yu, J. Zhao, J. Wang, S. Zheng, S. Lin, L. Chen, M. Yang, S. Jia, X. Zhang, P. Chen. *Palladium-triggered deprotection chemistry for protein activation in living cells*. *Nat. Chem*. *6*, 352-61 (**2014**).
